# A microRNA-based liquid biopsy signature for the early detection of esophageal squamous cell carcinoma: a retrospective, prospective and multicenter study

**DOI:** 10.1186/s12943-022-01507-x

**Published:** 2022-02-11

**Authors:** Jinsei Miyoshi, Zhongxu Zhu, Aiping Luo, Shusuke Toden, Xuantong Zhou, Daisuke Izumi, Mitsuro Kanda, Tetsuji Takayama, Iqbal M. Parker, Minjie Wang, Feng Gao, Ali H. Zaidi, Hideo Baba, Yasuhiro Kodera, Yongping Cui, Xin Wang, Zhihua Liu, Ajay Goel

**Affiliations:** 1 Center for Gastrointestinal Research; Center for Translational Genomics and Oncology, Baylor Scott & White Research Institute and Charles A. Sammons Cancer Center, Baylor University Medical Center, Dallas, TX USA; 2 Department of Gastroenterology and Oncology, Tokushima University Graduate School of Biomedical Sciences, Tokushima, Japan; 3 Department of Gastroenterology, Kawashima Hospital, Tokushima, Japan; 4 Department of Surgery, The Chinese University of Hong Kong. Prince of Wales Hospital, Shatin, N.T., Hong Kong, SAR China; 5 Department of Biomedical Sciences, City University of Hong Kong, Hong Kong, SAR China; 6 State Key Laboratory of Molecular Oncology, National Cancer Center/National Clinical Research Center for Cancer/Cancer Hospital, Chinese Academy of Medical Sciences and Peking Union Medical College, Beijing, 100021 China; 7 Department of Gastroenterological Surgery, Graduate School of Medical Sciences, Kumamoto University, Kumamoto, Japan; 8 Department of Gastroenterological Surgery, Nagoya University Graduate School of Medicine, Nagoya, Japan; 9 Division of Medical Biochemistry and Structural Biology, Institute of Infectious Disease and Molecular Medicine, University of Cape Town, Cape Town, South Africa; 10 Department of Clinical Laboratory, Chinese Academy of Medical Sciences and Peking Union Medical College, Beijing, China; 11 The Sixth Affiliated Hospital, Sun Yat-sen University, Guangzhou, China; 12 Esophageal and Lung Institute, Allegheny Health Network, Pittsburgh, PA USA; 13 Cancer Institute, Shenzhen Bay Laboratory, Shenzhen, China; 14 Cancer Institute, Peking University Shenzhen Hospital, Shenzhen Peking University-Hong Kong University of Science and technology (PKU-HKUST) Medical Center, Shenzhen, China; 15 Department of Molecular Diagnostics and Experimental Therapeutics, Beckman Research Institute of City of Hope, Duarte, CA USA; 16 City of Hope Comprehensive Cancer Center, Duarte, CA USA

**Keywords:** Cancer, Liquid biopsy, Biomarker, Esophageal squamous cell carcinoma, microRNA

## Abstract

**Background:**

Currently, there is no clinically relevant non-invasive biomarker for early detection of esophageal squamous cell carcinoma (ESCC). Herein, we established and evaluated a circulating microRNA (miRNA)-based signature for the early detection of ESCC using a systematic genome-wide miRNA expression profiling analysis.

**Methods:**

We performed miRNA candidate discovery using three ESCC tissue miRNA datasets (*n* = 108, 238, and 216) and the candidate miRNAs were confirmed in tissue specimens (*n* = 64) by qRT-PCR. Using a serum training cohort (*n* = 408), we conducted multivariate logistic regression analysis to develop an ESCC circulating miRNA signature and the signature was subsequently validated in two independent retrospective and two prospective cohorts.

**Results:**

We identified eighteen initial miRNA candidates from three miRNA expression datasets (*n* = 108, 238, and 216) and subsequently validated their expression in ESCC tissues. We thereafter confirmed the overexpression of 8 miRNAs (miR-103, miR-106b, miR-151, miR-17, miR-181a, miR-21, miR-25, and miR-93) in serum specimens. Using a serum training cohort, we developed a circulating miRNA signature (AUC:0.83 [95%CI:0.79–0.87]) and the diagnostic performance of the miRNA signature was confirmed in two independent validation cohorts (*n* = 126, AUC:0.80 [95%CI:0.69–0.91]; and *n* = 165, AUC:0.89 [95%CI:0.83–0.94]). Finally, we demonstrated the diagnostic performance of the 8-miRNA signature in two prospective cohorts (*n* = 185, AUC:0.92, [95%CI:0.87–0.96]); and (*n* = 188, AUC:0.93, [95%CI:0.88–0.97]). Importantly, the 8-miRNA signature was superior to current clinical serological markers in discriminating early stage ESCC patients from healthy controls (*p* < 0.001).

**Conclusions:**

We have developed a novel and robust circulating miRNA-based signature for early detection of ESCC, which was successfully validated in multiple retrospective and prospective multinational, multicenter cohorts.

**Supplementary Information:**

The online version contains supplementary material available at 10.1186/s12943-022-01507-x.

## Background

Esophageal cancer is the sixth leading cause of cancer-related deaths, and the eighth most common cancer worldwide, with a higher prevalence in specific geographical locations and certain ethnicities [1, 2]. Esophageal squamous cell carcinoma (ESCC) accounts for almost 80% of all esophageal cancer cases worldwide, with particularly high incidence rates in Eastern Asia and several regions of Africa [3]. The average 5-year survival rate for ESCC varies between 10 and 41% [4]. Such a poor prognosis stems from the presence of an extensive lymphatic network in the esophagus, compounded by the lack of a protective serosa, leading to aggressive early regional tumor advancement and metastasis. Furthermore, at early stages, ESCC patients are generally asymptomatic, resulting in delayed diagnosis [5]. Although the efficacy of various blood-based biomarkers (e.g., squamous cell carcinoma antigen [SCC-Ag], carcinoembryonic antigen [CEA] and cytokeratin-19 fragment [CYFRA21-1]) have been examined, none of these biomarkers are adequate as stand-alone ESCC diagnostic biomarkers [5, 6]. Therefore, there is an imperative need to develop reliable, non-invasive biomarkers for early detection of ESCC, which will play a pivotal role in improving patient outcomes.

MicroRNAs (miRNAs) are a class of small non-coding RNAs, approximately 20–25 nucleotides in length, that regulate gene expression through transcriptional interference or translational inhibition of downstream target genes (mRNAs). miRNAs are involved in most biological events, including tumorigenesis in the majority of human cancers, including ESCC [7]. Due to their stability and high abundance in bodily fluids, as well as their unique expression profiles under various biological conditions, circulating miRNAs are emerging as attractive candidates for non-invasive ‘liquid biopsy’ approaches [8, 9]. However, although several individual circulating miRNAs have been proposed for use in ESCC diagnosis [10, 11], their clinical translation potential remains questionable; primarily, due to limitations such as inadequate sensitivity and specificity of individual miRNAs, and their inability to account for tumor heterogeneity associated with ESCC [12].

Recent advances in RNA sequencing technologies have opened a new era of transcriptome-wide biomarker discovery, which enables in-depth molecular characterization of various cancers, including ESCC [13, 14]. The availability of large, multicenter, high-throughput datasets, together with unbiased, transcriptome-wide bioinformatic analysis, have paved the path for identification of more precise and robust molecular biomarker targets [15, 16]. Herein, we established a novel, non-invasive, miRNA-based signature using a systematic and comprehensive effort and by integrating transcriptome-wide biomarker discovery and clinical validations using 7 independent, retrospective and prospective, multinational, multicenter cohorts. Our 8-miRNA signature demonstrated considerable clinical value for the non-invasive detection of early stage ESCC patients, remarkably superior to conventional tumor biomarkers for ESCC [17]. Application of our circulating, epigenetic signature as a non-invasive, inexpensive and facile diagnostic assay for ESCC could improve the mortality of patients with ESCC, long considered one of the deadliest malignancies.

## Methods

### Study design

We analyzed approximately 1800 tissue and serum specimens from patients with ESCC, adjacent normal tissues and healthy participants in a five-phase study, which involved a biomarker discovery phase, a tissue validation phase, a retrospective serum validation phase, and a prospective serum performance evaluation phase (Fig. S1).

#### In-silico discovery phase

Three tissue-based genome-wide miRNA expression datasets (TCGA ESCC, GSE55856, and GSE43732) were used for the discovery of robust miRNA candidates. Significantly overexpressed miRNAs in cancer tissues were first identified from each dataset.

#### Tissue validation phase

The expression levels of the candidate miRNAs identified in the discovery phase were evaluated using qRT-PCR in matched tumor and adjacent normal tissues from 32 ESCC patients collected from Nagoya University Hospital, Nagoya, Japan during 2001 and 2015.

#### Retrospective serum biomarker prioritization phase

To develop a circulating miRNA signature, we assessed expression of the candidate miRNAs in an age-, sex-, and race-matched serum cohort of 50 ESCC patients and 50 healthy controls. These samples were collected from the Kumamoto University Hospital, Japan enrolled between 2009 and 2011.

#### Retrospective serum training and validation phase

Using the miRNAs that were prioritized in the previous step as covariates, multivariate logistic regression analysis was employed to establish an ESCC risk-scoring formula using qRT-PCR data available from the serum training cohort (*n* = 408). These samples were collected from the Groote Schuur Hospital, Cape Town, South Africa between 2001 and 2015. The diagnostic performance of the 8-miRNA signature was thereafter evaluated in serum validation cohort 1 (*n* = 126) (Kumamoto University Hospital between 2012 and 2016) and serum validation cohort 2 (*n* = 165) (Nagoya University Hospital between 2001 and 2015).

#### Prospective serum training and validation phase

In order to prospectively examine the circulating miRNA signature, serum specimens were collected from 178 patients with ESCC and 195 healthy individuals, matched by age and sex, who were prospectively recruited from February to July 2018 at the National Cancer Center/National Clinical Research Center for Cancer/Cancer Hospital, Chinese Academy of Medical Sciences, Beijing, China (Table 1). qPCR quantification was performed on the Beijing-1 cohort (89 ESCC vs. 96 healthy). The data generated from the Beijing-1 cohort was used to train a multivariate logistic regression model and establish an ESCC risk-scoring formula. The performance of the circulating miRNA signature was subsequently evaluated based on qPCR data from the Beijing-2 cohort (89 ESCC vs 99 healthy).

**Table 1:** Clinical characteristics of patients and healthy participants in the tissue validation, and retrospective and prospective serum cohorts

	Tissue Cohort	Serum Cohorts (Retrospective)				Serum Cohorts (Prospective)	
	Validation Cohort (***n*** = 64)	Prioritization Cohort (***n*** = 100)	Training Cohort (***n*** = 408)	Validation Cohort 1 (***n*** = 126)	Validation Cohort 2 (***n*** = 165)	Training Cohort (***n*** = 185)	Validation Cohort (***n*** = 188)
ESCC	32 (50)	50 (50)	280 (68.6)	106 (84.1)	123 (74.5)	89 (48.1)	89 (47.3)
Sex							
Men	26 (81.2)	29 (58)	188 (67.1)	66 (62.3)	93 (75.6)	79 (88.8)	78 (87.6)
Women	6 (18.8)	21 (42)	92 (32.9)	40 (37.7)	30 (24.4)	10 (11.2)	11 (12.4)
Age, median (range), y	60 (54–77)	55 (35–70)	59 (28–87)	56 (30–76)	65 (44–84)	62 (55–67)	62 (55–67)
Cancer stage							
I	6 (18.8)	21 (42)	8 (2.8)	43 (40.6)	22 (17.9)	12 (13.5)	13 (14.6)
II	10 (31.2)	11 (22)	61 (21.8)	23 (21.7)	30 (24.4)	19 (21.3)	20 (22.5)
III	16 (50)	18 (36)	180 (64.3)	40 (37.7)	61 (49.6)	25 (28.1)	26 (29.2)
IV			21 (7.5)		10 (8.1)	31 (34.8)	30 (33.7)
Unstaged			10 (3.6)			2 (2.3)	
Differentiation							
Well (W)						8 (9.0)	11 (12.4)
Moderate (M)						23 (25.8)	27 (30.3)
P (Poor)						16 (18.0)	16 (18.0)
Unknown						42 (47.2)	35 (39.3)
Location							
Lower (L)						38 (42.7)	32 (35.9)
Middle (M)						14 (15.7)	16 (18.0)
Upper (U)						14 (15.7)	11 (12.4)
Unknown						23 (25.9)	30 (33.7)
Race							
Asian	32 (100)	50 (100)		106 (100)	123 (100)	89 (100)	89 (100)
Black			180 (64.3)				
Mixed-race			100 (35.7)				
**Healthy participants**	32 (50)	50 (50)	128 (31.4)	20 (15.9)	42 (25.5)	96 (51.9)	99 (52.7)
Sex							
Men		31 (62)	88 (68.8)	11 (55)	23 (54.8)	63 (65.6)	65 (65.7)
Women		19 (38)	40 (31.2)	9 (45)	19 (45.2)	33 (34.4)	34 (34.3)
Age, median (range), y		54 (33–66)	55 (30–76)	53 (35–64)	37 (26–56)	57 (50–65)	57 (48–64)
Race							
Asian	32 (100)	50 (100)		20 (100)	42 (100)	96 (100)	99 (100)
Black			81 (63.3)				
Mixed-race			47 (36.7)				

Detailed information on cohorts is provided in the Supplementary Materials.

### Sample preparation

Tissue samples (tumor and the corresponding normal mucosa) were obtained from patients submitted to esophagectomy without any pre-operative therapy and were immediately placed in RNAlater (Qiagen, Germany), then stored at − 80 °C. Whole blood samples from each participant were collected before treatment and subjected to centrifugation at 3000 g for ten minutes within 12 h after collection. The resulting serum samples were stored in RNase-free Eppendorf tubes at − 80 °C.

### RNA isolation

RNA was isolated from tissue specimens using the RNeasy Mini Kit (Qiagen). RNA was eluted in 30 μL of RNase-free water using a QIAcube semiautomated robotic device (Qiagen), quantified using a NanoDrop spectrophotometer (NanoDrop Technologies, Wilmington, DE), and stored at − 80 °C until further use. For serum RNA isolation, miRNAeasy Serum/Plasma Kit (Qiagen) was used to extract RNA enriched in small RNAs. Briefly, serum samples were thawed on ice and centrifuged at 10,000 rpm for 5 minutes to remove cellular debris. Two hundred μL of supernatant was lysed in 1000 μL of Qiazol Lysis Reagent. For normalization of sample-to-sample variation during the RNA isolation procedures, 25 fmol of synthetic *C. elegans* miRNA (cel-miR-39, Qiagen) was added to each 200 μl denatured sample [18]. Total RNA, including small RNA, was extracted and eluted in 30 μL of RNase-free water using a QIAcube semiautomated robotic device (Qiagen) and stored at − 80 °C for further use.

### Quantitative reverse transcription polymerase chain reaction (qRT-PCR)

For miRNA-based qRT-PCR assays, 1.2 μL of RNA from tissue/serum samples was reverse-transcribed using the TaqMan MicroRNA Reverse Transcription Kit (Applied Biosystems, Carlsbad, CA) in a total reaction volume of 6 μL. Real-time PCR was conducted using MicroRNA Assay Kits and TaqMan Universal Master Mix II, no UNG (Applied Biosystems) using QuantStudio 6 Flex Real-Time PCR System (Applied Biosystems) QuantStudio DX system (Applied Biosystems) was used for the prospectively collected samples. The expression of miRNAs was normalized to U6 in tissue specimens (Ambion, Austin, TX) and to miR-16 in serum specimens [19] for retrospectively collected specimens and normalized to miR-16 and miR-423 for prospectively collected specimens (Applied Biosystems). All data are represented as 2^-ΔΔCt^.

### Cyfra-21, CEA, and SCC detection

Serum from healthy control and ESCC groups were used to assess circulating protein levels of Cyfra-21, and CEA by Cobas e 601 (Roche Diagnostics) and SCC using ABBOTT (i2000SR).

### Cost-effectiveness analysis

A cost-effectiveness analysis was performed under the following clinical assumptions: Non-invasive screening was performed on a high-risk population, Chinese men over 40 years old. The compliance rate was estimated to be approximately 45% [20]. The positive test group would go on to have a confirmatory test using endoscopy and biopsy. The biopsy test is considered a gold standard, with 100% sensitivity and specificity. The negative test group would go on to have a 3-year follow-up, during which cancer patients would be detected. For the non-screening group, 10% of the high-risk population was estimated to receive an endoscopy test to evaluate the incidence of cancer. Due to the high sensitivity and specificity of the 8-miRNA signature assay, we estimated that the rate of patients diagnosed at an early stage will improve in comparison to current conventional methods.

For the assumption of cancer treatment, early or advanced stages (TNM Stage 1–3) were considered curable and it was assumed that patients would be cured after 2 years with a stage-specific recurrence rate. Terminal stage (TNM Stage 4) was considered as untreatable, with only palliative care and death after 1 year. Considering that cancer recurrence is associated with poor prognosis, all relapsed patients were assumed to have Stage 4 status. Cost and incidence rate were either collected from the literature or estimated by our in-house clinical records (Table S10).

### miRNA regulatory network inference and functional analysis

A miRNA–mRNA network was constructed to study the regulatory functions of the candidate miRNAs. More specifically, for each of the miRNAs, its target mRNAs were identified based on the following criteria: 1) the miRNA–mRNA interactions had been experimentally validated based on the miRTarBase database (V8); 2) the mRNAs were differentially expressed between tumor and normal samples (|log2 fold change| > 2 & Benjamini-Hochberg (BH)-adjusted *p* < 0.01) in the TCGA dataset [21]. Functional analysis was performed based on hypergeometric tests using the “clusterProfiler” package, with KEGG pathways and cancer Hallmark gene sets retrieved from the MSigDB Database (v7.0) [22–24]. *P*-values were corrected for multiple hypothesis testing using the BH Procedure, and BH-adjusted *p* < 0.05 was considered statistically significant.

### Statistical analysis

Differential miRNA expression between paired groups, as well as two independent groups, was analyzed using two-sided student’s *t*-tests, in which a *p*-value of < 0.05 was considered statistically significant. A receiver operating characteristic (ROC) curve was generated and the area under the ROC curve (AUC) was computed with 95% confidence intervals (CI) to assess the discriminative performance of a miRNA. Multivariate logistic regression was employed to derive a formula to predict ESCC risk. All statistical analyses were performed using Medcalc statistical software (v.12.7.7., Medcalc Software bvba, Ostend, Belgium), JMP software (10.0.2., SAS Institute, Cary, NC, USA), and R (3.3.3, R Development Core Team, https://cran.r-project.org/).

## Results

### Study design and characteristics of ESCC patients and control participants

Our study design consisted of five major phases: an *in-silico* discovery phase, a tissue validation phase, a retrospective serum biomarker prioritization phase, a retrospective serum training and validation phase, and a prospective serum training and validation phase (Fig. S1). The characteristics of all study participants whose samples we used to develop and validate a miRNA signature are summarized in Table 1.

### Identification of an 18-miRNA panel that robustly discriminates ESCC from normal mucosa

**Fig. 1:**
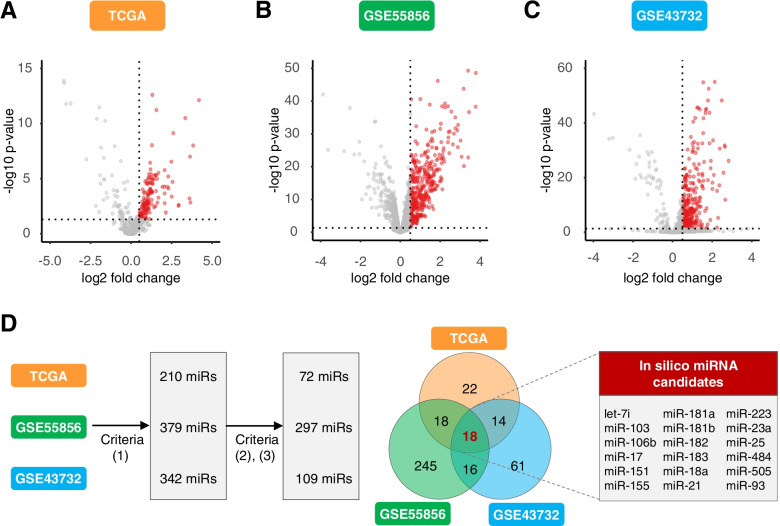
Genome-wide discovery of miRNA candidates for ESCC diagnosis in tissue. Volcano plots for three independent miRNA expression datasets: TCGA (**A**), GSE55856 (**B**) and GSE43732 (**C**). **D** 18 candidates miRNAs were identified by overlapping strategy

In the discovery phase, we first interrogated three transcriptome-wide tissue-based miRNA expression profiling datasets (TCGA, GSE55856, and GSE43732) to prioritize miRNA candidates. We considered a miRNA to be a potential candidate if it was: (1) differentially expressed between ESCC and normal tissue (criteria: log2 fold-change> 0.5, FDR-adjusted *p* < 0.05); (2) discriminative between ESCC and normal specimens (criteria: AUC > 0.7); (3) upregulated in ESCC, with a relatively high expression to facilitate detection in serum samples (criteria: average expression > median average expression of all differentially expressed miRNAs). Consequently, we identified 72, 297, and 109 miRNAs from the TCGA, GSE55856, and GSE43732 datasets, respectively (Fig. 1A-C). Comparison between three data sets resulted in identification of a panel of 18 miRNAs that overlapped between all three expression datasets (Fig. 1D), which was selected for subsequent analysis. The principal component analysis showed that these miRNAs resulted in the formation of distinct clusters between ESCC patients and healthy individuals suggesting that these miRNAs could be used to discriminate ESCC patient (Fig. S2).

**Fig. 2:**
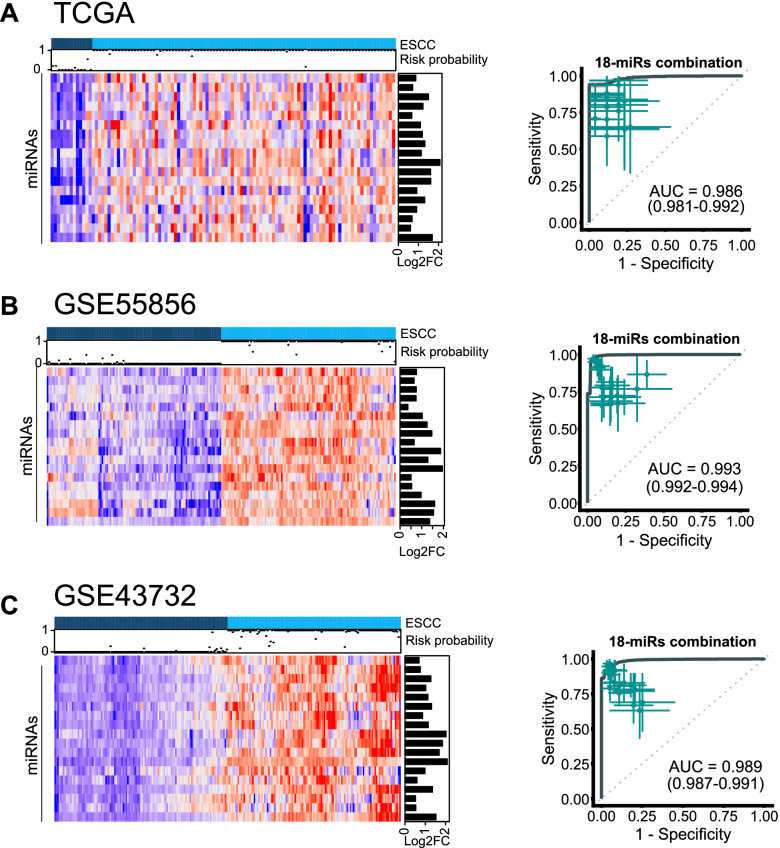
The diagnostic performance of 18-mRNA signature for distinguishing cancer and normal tissues. Heatmaps for TCGA (**A**), GSE55856 (**B**) and GSE43732 (**C**), respectively. Heatmaps illustrate expression of the 18 candidate miRNAs in the three miRNAs expression datasets. The upper panel show the risk probabilities derived from multivariate regression analysis with 2-fold cross-validation (repeated 100 times), and the right panel showed the expression fold changes of the 18 candidate miRNAs. The ROC curves demonstrate that the 18-miRNA signature accurately distinguished cancer tissues from normal tissues in all three datasets (average AUC = 0.986, 0.993, 0.989, for TCGA (**A**), GSE55856 (**B**), and GSE43732 (**C**) respectively), and superior to single panel member. ROC curve is shown with 95% CI. The 95% CI of sensitivity and specificity for each panel member was also shown at the best threshold (calculated by Youden-Index)

To evaluate the diagnostic potential of the 18-miRNA panel, we employed a two-pronged strategy. First, within each dataset, we performed multivariate logistic regression with 2-fold cross-validations (repeated 100 times) to demonstrate the diagnostic performance of the signature (average AUC = 0.98, 0.99, 0.98, respectively; Fig. 2). Second, we trained a multivariate logistic regression model on the GSE55856 dataset, and then applied the same statistical model to all three datasets in order to further validate and confirm the diagnostic performance of the 18-miRNA signature (AUC = 0.99, 1.00, 0.99, respectively; Fig. S3).

Next, to determine the functional significance of these candidate miRNAs, we constructed a miRNA–mRNA regulatory network based on experimentally validated miRNA–target interactions obtained from the miRTarBase database (V8). In total, we identified 393 genes that were differentially expressed between tumor and normal samples in the TCGA dataset based on the following criteria, |log2 fold-change| > 2 and BH-adjusted *p* < 0.01, as targets of the 18 miRNAs (Fig. S4A, Table S2). As expected, these miRNA target genes were significantly enriched in cancer-related signaling pathways, such as epithelial-to-mesenchymal transition and angiogenesis pathways (Fig. S4B, Table S3). To ensure that these 18 miRNAs are dysregulated in ESCC, we analyzed a cohort of 32 ESCC and 32 matched adjacent normal tissue specimens to confirm the upregulation of all 18 miRNAs in ESCC (*p* < 0.05, paired student *t*-tests; Fig. S5); highlighting their diagnostic significance and biological relevance in esophageal cancer.

### Training and validation of an 8-miRNA circulating signature in serum from retrospective cohorts of ESCC patients

**Fig. 3:**
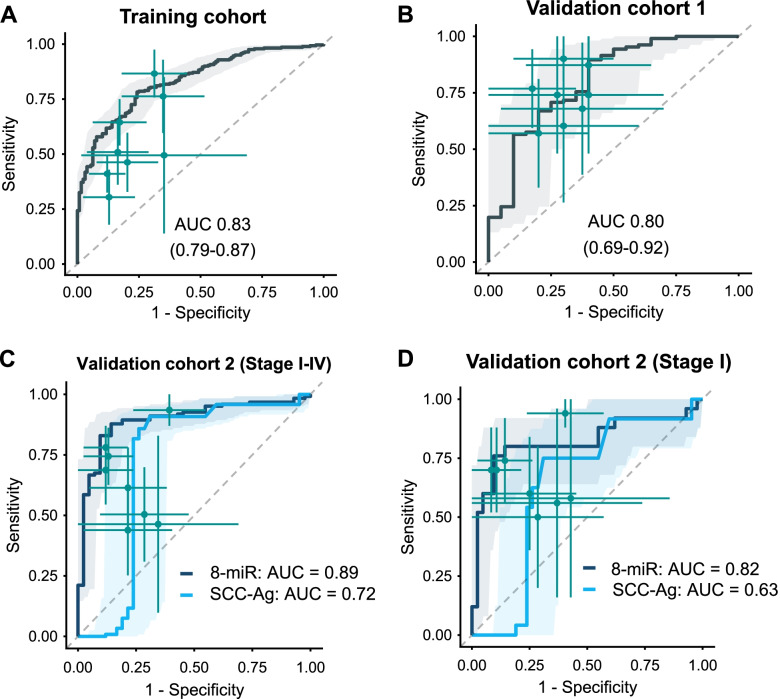
Establishment, validation, and diagnostic performance evaluation of an 8-miRNA signature. ROC curves were used to demonstrate the robust diagnostic value of the 8-miRNA signature in (**A**) the serum training cohort (AUC = 0.83), (**B**) the validation cohort 1 (AUC = 0.80), (**C**) stage I–IV patient samples of validation cohort 2 (AUC = 0.89), and (**D**) only stage I samples of validation cohort 2 (AUC = 0.82). CI was calculated by 2000 stratified bootstrap replicates

Considering that our aim was to develop a non-invasive liquid biopsy assay, we next examined the diagnostic performance of the tissue-based 18-miRNA panel for its translational potential in a serum-based biomarker prioritization cohort (50 ESCC, 50 healthy controls). Among the 18 miRNAs, the expression levels of four miRNAs (miR-182, miR-183, miR-18a and miR-505) were below the detection limit in serum specimens (average PCR cycle threshold > 35; Table S4) [25]. For the remaining 14 miRNAs, eight (miR-103, miR-106b, miR-151, miR-17, miR-181a, miR-21, miR-25 and miR-93) were significantly upregulated in serum from ESCC patients compared to healthy controls (*p* < 0.05, student *t*-tests; Fig. S6). From a clinical standpoint, measurement of an upregulated marker in blood is more practical, therefore we focused on 8 upregulated miRNA candidate and interrogated the diagnostic performance of the 8-miRNA panel in training cohort of patients (280 ESCC, 128 healthy controls), which allowed us to construct a multivariate logistic regression model (Table S4). We subsequently derived a risk-scoring formula using logistic regression. For all retrospective serum cohorts, we used this scoring formula and Youden’s index (0.582) derived from the serum training cohort as the cutoff thresholds to dichotomize high- and low-risk groups. Using the risk-scoring formula and the cutoff values, we evaluated the diagnostic performance of this 8-miRNA panel in the training cohort by means of AUC and corresponding 95% confidence intervals, sensitivity, and specificity. Interestingly, for the serum training cohort, this miRNA signature achieved an AUC of 0.83 (95% CI, 0.79–0.87), a sensitivity of 78%, and a specificity of 75% (Fig. 3A and S7A).

To further confirm the diagnostic performance of this 8-miRNA signature, we assessed its performance in two additional independent patient cohorts, where we were able to collect serum specimens – the serum validation cohort 1 (106 ESCC patients and 20 healthy controls) and serum validation cohort 2 (123 ESCC patients and 42 healthy controls). Consistent with the serum training cohort, our circulating miRNA signature achieved a robust performance in both serum validation cohort 1 (Fig. 3B, S7B, AUC = 0.80, 95% CI: 0.69–0.91, sensitivity: 89%, specificity: 60%) and serum validation cohort 2 (Fig. 3C, S7C, S8, Table S5, AUC = 0.89, 95% CI: 0.83–0.94, sensitivity: 87%, specificity: 85%).

Next, using the serum validation cohort 2, we compared the diagnostic performance of our 8-miRNA signature against that of a classic tumor marker in ESCC patients – the squamous cell carcinoma-related antigen (SCC-Ag). While the SCC-Ag levels exhibited modest diagnostic efficiency (Fig. 3C, AUC = 0.72, 95% CI: 0.60–0.84, sensitivity: 0.91, specificity: 0.69), our 8-miRNA panel was significantly superior in distinguishing ESCC patients across all disease stages (Fig. 3C, *p* = 0.003, DeLong’s test). Furthermore, even when we evaluated specifically in stage I ESCC patients, our circulating miRNA signature maintained its diagnostic performance in discriminating stage I ESCC patients (*n* = 20) from healthy controls (*n* = 42; AUC = 0.82, 95% CI:0.70–0.94, sensitivity: 0.76, specificity: 0.91). Likewise, our biomarker panel also maintained its superiority to SCC-Ag in the stage I patients as well (*p* = 0.025, DeLong’s test; AUC = 0.63, 95% CI: 0.50–0.78, sensitivity: 0.75, specificity: 0.69; Fig. 3D, Table S5), highlighting its potential as a promising early diagnostic assay.

To investigate whether our 8-miRNA panel has a diagnostic specificity for ESCC and not other cancer types, we evaluated the diagnostic performance of our 8-miRNA panel in other major malignancies including colorectal, prostate, lung and breast cancer using public serum miRNA datasets. The scoring formula of the 8-miRNA panel was applied to these datasets and the diagnostic performance of the panel discriminating cancer patients from healthy controls in each cancer types was evaluated. Compared to ESCC, the AUC values of the 8-miRNA panel substantially decreased in other cancer types (combined ESCC validation cohorts VS. other cancer types, all *P* < 0.05, DeLong’s tests, Fig. S9), suggesting that our 8-miRNA panel is specific to ESCC. Collectively, these data support the diagnostic efficacy of the 8-miRNA signature, as well as its promising potential for the detection of early stage ESCC.

### Validation of the diagnostic performance of the circulating miRNA signature in two, independent, prospective cohorts of ESCC patients

**Fig. 4:**
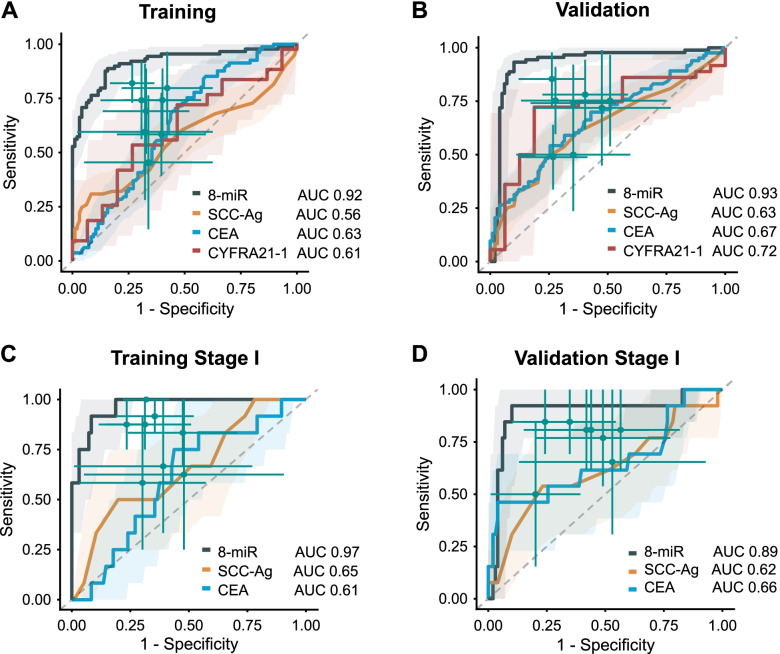
Evaluation of the circulating miRNA signature for detection of ESCC in randomized prospective cohorts. ROC curves were generated to assess the diagnostic performance of the 8-miRNA signature in both (**A**) Beijing-1 (AUC = 0.92), and (**B**) Beijing-2 (AUC = 0.93) randomized prospective cohorts (ESCC patients across stages). Compared to our 8-miRNA signature, CE72–4, cyfra21–1, SCC-Ag, and CEA markers all showed significantly poorer performance (all *P* < 0.01, DeLong’s tests) in both cohorts. CI was calculated by 2000 stratified bootstrap replicates. Compared to conventional SCC-Ag and CEA markers, our 8-miRNA signature also demonstrated its superior performance in detection of stage I ESCC patients in both (**C**) the Beijing-1 cohort (AUC = 0.97, all *P* < 0.05, DeLong’s tests) and (**D**) the Beijing-2 cohort (AUC = 0.89, all P < 0.05, DeLong’s tests)

To demonstrate the clinical application of our circulating miRNA signature in true clinical settings, we next examined its performance in two, randomized, prospectively enrolled patient cohorts. We performed qRT-PCR assays to assess the expression of the 8-miRNAs signature in 186 serum specimens (Beijing-1 cohort; 84 ESCC patients and 102 healthy controls) and used this cohort as our training set. We performed multivariate logistic regression analysis and derived a risk-scoring formula: logit(P) = (0.00810 x miR17)–(0.183 x miR21)–(0.974 x miR25) + (0.973 x miR93)–(0.347 x miR103)–(0.298 x miR106b)-(0.194 x miR151) + (0.226 x miR181a)-3.196. Our 8-miRNA signature performed robustly in its ability to distinguish ESCC patients from healthy controls (Fig. 4A, S10A, S11A, AUC = 0.92, 95% CI: 0.87–0.96, sensitivity: 89%, specificity: 84%). Subsequently, we assessed the performance of this miRNA signature in an independent validation cohort (Beijing-2 cohort; 89 ESCC patients and 99 healthy controls). Once again, our signature robustly distinguished ESCC patients from healthy controls (Fig. 4B, S10B, S11B, S12, AUC = 0.93, 95% CI: 0.88–0.97, sensitivity: 93%, specificity: 89%; Table S6). In both training and validation cohorts, our 8-miRNA signature performed substantially better than individual miRNAs in identifying ESCC patients (S11A and S11B).

In both cohorts, compared to the conventional tumor markers including SCC-Ag, CEA, and CYFRA21-1, our 8-miRNA panel consistently demonstrated superior diagnostic performance for the identification of ESCC patients across all stages (Table S6, Fig. 4A, B, all *p* < 0.01, DeLong’s tests). Notably, when we focused on stage I ESCC patients, our 8-miRNA signature remarkably discriminated stage I ESCC patients from healthy controls in both Beijing-1 cohort (AUC = 0.97, 95% CI:0.93–1.00, sensitivity: 0.92, specificity: 0.92) and Beijing-2 cohorts (AUC = 0.89, 95% CI: 0.77–1.00, sensitivity: 92%, specificity: 90%); and in each instance its performance was substantially superior to that of SCC-Ag and CEA, which are routinely analyzed in clinical settings (Table S7, Fig. 4C and D, all *p* < 0.05, DeLong’s tests). We performed univariate and multivariate analyses to confirm that our circulating miRNA signature was the only significant predictor for detecting ESCC patients from all stages (Table S8), as well as stage I patients specifically (Table S9).

### The 8-miRNA signature robustly identifies patients with high-risk premalignant lesions and is cost-effective vs. currently used diagnostic approaches in the clinic

**Fig. 5:**
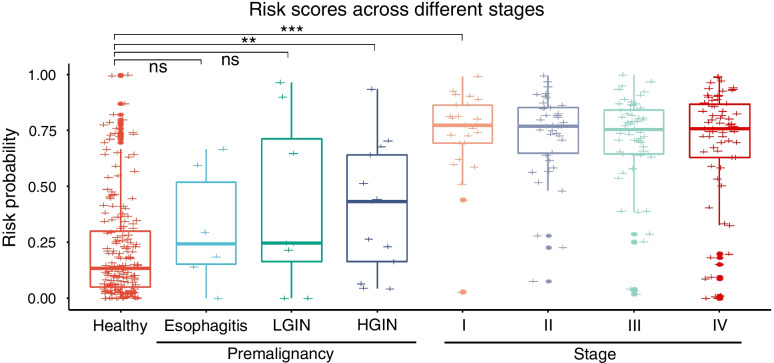
The miRNA-classifier effectively discriminates stage I ESCC and premalignant lesions. Boxplots comparing risk scores between ESCC of different stages, premalignant lesions (esophagitis, low-grade intraepithelial neoplasia [LGIN], and high-grade intraepithelial neoplasia [HGIN]) and healthy controls. ** P < 0.01, *** *P* < 0.001

Next, we investigated the earliest possible lesions that could be detected with our non-invasive circulating miRNA panel. Since the diagnostic risk scores were significantly elevated in stage I–IV ESCC patients (all *p* < 0.001, one-sided Student’s *t*-tests), we examined the diagnostic performance of the 8-miRNA panel for identifying patients with high-grade intraepithelial neoplasia. Intriguingly, the panel was able to identify patients with high-grade intraepithelial neoplasia (*n* = 13, *p* < 0.01, one-sided Student’s *t*-test; Fig. 5). However, the risk scores did not change significantly in patients with low-grade intraepithelial neoplasia (*n* = 8) or those with esophagitis (*n* = 6) compared to healthy controls (Fig. 5). These results suggest a potential use of our circulating miRNA signature for early detection of high-risk premalignant lesions.

To determine whether screening using our miRNA signature would be cost effective, we performed cost effective analysis (see Supplementary Material for details). We estimated mass screening using our circulating miRNA signature to be cost-effective relative to current practice [ICER = CNY 15,800.4/QALY] (Tables S10, S11). In summary, our circulating miRNA signature demonstrated promising diagnostic performance in our multinational, multicenter cohort study, and is likely to provide a cost-efficient, highly robust option for non-invasive early detection of ESCC.

## Discussion

ESCC is one of the most aggressive cancers and its low patient survival rate is primarily due to delayed diagnosis [26]. Therefore, early detection of ESCC provides opportunities to implement effective treatment strategies and timely interventions to improve patients’ overall outcomes. However, there is currently no clinically viable molecular marker for non-invasive diagnosis of ESCC. In this study, we performed a comprehensive bioinformatics analysis to identify candidate miRNAs from three in silico datasets and subsequently developed a panel of 8 circulating miRNAs for non-invasive ESCC detection. We demonstrated the diagnostic performance of the miRNA diagnostic panel in several large, independent, retro-prospective, multinational, multicenter cohorts.

Both genetic and epigenetic changes are recognized as the key contributors in cancer development. miRNAs have been recognized as promising non-invasive biomarker candidates, primarily due to their structural stability and abundance in circulation [27]. Accordingly, a plethora of studies has examined the diagnostic potential of circulating miRNAs in various cancers, including ESCC [11, 28]. While epigenetic alterations occur more frequently at an early stages of cancer development, mutations in p53, the most frequently occurring mutations in ESCC, have been shown to modulate the expression levels of miRNAs [29].

In ESCC, the expression of several circulating miRNAs has been evaluated individually for ESCC diagnosis and several studies have attempted to combine multiple miRNAs to establish a miRNA-based ESCC diagnostic panel [11, 30, 31]. However, the diagnostic potential of individual circulating miRNA markers was limited, and the panels derived for the detection of ESCC were constructed with poor or biased candidate selection criteria and lacked validation in multiple cohorts. Although these studies highlight the clinical usefulness of circulating miRNAs, the above limitations result in poor data interpretation. Furthermore, although ethnicity and geographical distribution play a major role in ESCC incidence [3], previous studies did not account for such variations when assessing the diagnostic performance of their miRNA markers. In this study, we successfully established systematic, comprehensive, and reliable biomarker discovery approach, using numerous global, multicenter, and retro-prospective cohorts of more than 1800 clinical specimens. To our knowledge, we tested our panel using the largest and most ethnically and geographically diverse ESCC sample collection to date. In addition, we showed that the miRNA panel had a significantly superior detection capability compared to conventional clinical serological markers, including SCC-Ag, the most commonly used serum diagnostic marker for ESCC [32]. We also showed using multiple cancer datasets that our miRNA panel was specific to ESCC diagnosis and not other cancer types. Furthermore, the strongest point of our study is that we expanded evaluation of our miRNA panel to prospectively collected samples to accurately assess its diagnostic performance. Although our 8-miRNA signature demonstrated effectiveness regardless of race (i.e., in two Asian and one African cohort) in our retrospective validation, it is important note that the diagnostic classifiers were developed using primarily Asian cohorts. Therefore, future studies are needed to optimize performance of the risk-scoring model in additional prospective serum cohorts and test the diagnostic performance of the classifiers in cohorts comprised of non-Asian races. Another potential limitation of our study is that we prioritized miRNA biomarkers that were overexpressed in ESCC tissues, with the hypothesis that such miRNAs are the most likely to be released into systemic circulation. However, recent studies have indicated that some miRNAs that do not accumulate in tissues may still be excreted in extracellular-vesicles such as exosomes [33, 34]. In addition, although our diagnostic miRNA panel was robust in identified ESCC patients, we acknowledge that a portion of patients had false positive outcomes. Lastly, given that the primary focus of our present study was development of a diagnostic assay for ESCC, we are unable to determine whether these markers could also predict response to treatment in ESCC patients as well – an important consideration that will pursue in subsequent studies. Based on the cancer screening biomarker pipeline [35], we plan to perform a retrospective performance study [36] to evaluate the diagnostic performance of the miRNA signature.

In conclusion, we used a comprehensive biomarker discovery process with three large independent public datasets, one tissue cohort, and four retrospective and two prospective large independent serum cohorts to develop and successfully validate a novel and robust miRNA-based signature for the early detection of ESCC. While additional validation studies are required to comprehensively evaluate the performance of our classifiers, our miRNA signature has the potential to transform noninvasive diagnosis for ESCC patients in the future.

## Supplementary Information

**Additional file 1: Supplementary Figure 1.** Study design for the identification and validation of the circulating miRNA panel for ESCC detection. **Supplementary Figure 2.** 18-miRNAs can distinguish between tumor and normal tissues*.* **Supplementary Figure 3.** In silico validation for 18-miRNA panel and Silhouette analysis. **Supplemental Figure 4.** miRNA regulatory network analysis and functional analysis of miRNA target genes. **Supplemental Figure 5.** Tissue validation for initial miRNA candidates. **Supplemental Figure 6.** Selection of circulating miRNAs in the serum biomarker prioritization cohort. **Supplementary Figure 7**. The robustness of the miRNA-classifier in training and validation cohorts*.* **Supplemental Figure 8.** Confusion matrices analysis for validation cohort 2. **Supplemental Figure 9.** Specificity analysis for the 8-miRNA panel on multiple cancer types. **Supplemental Figure 10.** The robustness of the miRNA-classifier in the prospectively collected cohorts. **Supplemental Figure 11.** Specificity analysis for the 8-miRNA panel and individual miRNAs. **Supplemental Figure 12.** Confusion matrices analysis for the Beijing-2 prospective cohort. **Supplemental Table 1.** Characteristics of in silico discovery sets. **Supplemental Table 2.** miRNA–mRNA interactions in the regulatory network. **Supplemental Table 3.** Functional analysis of miRNA target genes identified 31 significantly enriched signaling pathways and Hallmark gene sets (BH-adjusted *p*-value < 0.05). **Supplemental Table 4.** miRNA panel selection and logistic regression model in serum biomarker prioritization and training phases. **Supplemental Table 5.** Prediction of serum 8-miR panel and serum SCC-Ag for the differential diagnosis of ESCC from healthy participants in serum training and serum validation cohorts. **Supplemental Table 6.** Comparison of the performance of the circulating miRNA signature against SCC-Ag, CEA, CA72–4, and CYFRA21-1 for non-invasive detection of ESCC across all stages in randomized prospective serum cohorts. **Supplemental Table 7.** Benchmark the performance of the circulating miRNA signature against SCC-Ag and CEA for non-invasive detection of stage I ESCC in randomized prospective serum cohorts. **Supplemental Table 8.** Univariate and multivariate analyses of the circulating miRNA signature with SCC-Ag, CEA, CA72–4, and CYFRA21-1 for non-invasive detection of ESCC across all stages in randomized prospective serum cohorts. **Supplemental Table 9.** Univariate and multivariate analyses of the circulating miRNA signature with SCC-Ag and CEA for non-invasive detection of stage I ESCC in randomized prospective serum cohorts. **Supplemental Table 10.** Results of cost-effectiveness analysis for non-invasive screening for Chinese men in China (> 40 years old). **Supplemental Table 11.** Base-case values in cost-effectiveness modeling.

## Data Availability

All data derived from public database are available from these sites. TCGA Research Network: http://cancergenome.nih.gov/ (ESCC dataset). Gene Expression Omnibus https://www.ncbi.nlm.nih.gov/geo/ (GSE55856, and GSE43732). All other data are available on reasonable request from the corresponding authors.

## Abbreviations

ESCC: Esophageal squamous cell carcinoma
miRNA: MicroRNA
TCGA: The Cancer Genome Atlas
AUC: Area under the curve
CI: Confidence interval
SCC: Squamous cell carcinoma
GEO: Gene expression omnibus
BC: Breast cancer
CRC: Colorectal cancer
PC: Prostate cancer
LC: Lung cancer
ROC: Receive operator characteristic curve
