# Supplementary material for: A microRNA-based liquid biopsy signature for the early detection of esophageal squamous cell carcinoma: a retrospective, prospective and multicenter study
Source: Mol Cancer. 2022 Feb 11;21:44. doi: 10.1186/s12943-022-01507-x (PMC8832722; doi:10.1186/s12943-022-01507-x)
Supplement: Supplementary file 1 — Additional file 1: Supplementary Figure 1. Study design for the identification and validation of the circulating miRNA panel for ESCC detection. Supplementary Figure 2. 18-miRNAs can distinguish between tumor and normal tissues. Supplementary Figure 3. In silico validation for 18-miRNA panel and Silhouette analysis. Supplemental Figure 4. miRNA regulatory network analysis and functional analysis of miRNA target genes. Supplemental Figure 5. Tissue validation for initial miRNA candidates. Supplemental Figure 6. Selection of circulating miRNAs in the serum biomarker prioritization cohort. Supplementary Figure 7. The robustness of the miRNA-classifier in training and validation cohorts. Supplemental Figure 8. Confusion matrices analysis for validation cohort 2. Supplemental Figure 9. Specificity analysis for the 8-miRNA panel on multiple cancer types. Supplemental Figure 10. The robustness of the miRNA-classifier in the prospectively collected cohorts. Supplemental Figure 11. Specificity analysis for the 8-miRNA panel and individual miRNAs. Supplemental Figure 12. Confusion matrices analysis for the Beijing-2 prospective cohort. Supplemental Table 1. Characteristics of in silico discovery sets. Supplemental Table 2. miRNA–mRNA interactions in the regulatory network. Supplemental Table 3. Functional analysis of miRNA target genes identified 31 significantly enriched signaling pathways and Hallmark gene sets (BH-adjusted p-value < 0.05). Supplemental Table 4. miRNA panel selection and logistic regression model in serum biomarker prioritization and training phases. Supplemental Table 5. Prediction of serum 8-miR panel and serum SCC-Ag for the differential diagnosis of ESCC from healthy participants in serum training and serum validation cohorts. Supplemental Table 6. Comparison of the performance of the circulating miRNA signature against SCC-Ag, CEA, CA72–4, and CYFRA21-1 for non-invasive detection of ESCC across all stages in randomized prospective serum cohorts. Supple [file 12943_2022_1507_MOESM1_ESM.docx]

Supplementary Materials

**A microRNA-based liquid biopsy signature for the early detection of esophageal squamous cell carcinoma: A retrospective, prospective and multicenter study**

Jinsei Miyoshi, MD, PhD^1,2,*^, Zhongxu Zhu, PhD^3,4,*^, Aiping Luo, PhD^5,*^, Shusuke Toden, PhD^1.*^, Xuantong Zhou, PhD^5^, Daisuke Izumi, MD, PhD^6^, Mitsuro Kanda, MD, PhD^7^, Tetsuji Takayama, MD, PhD^2^, Iqbal M Parker, PhD^8^, Minjie Wang, PhD^9^, Feng Gao, PhD^10^, Ali Zaidi, MD, PhD^11^, Hideo Baba, MD, PhD^6^, Yasuhiro Kodera, MD, PhD^7^, Yongping Cui, PhD^12,13^, Xin Wang, PhD^3,+^, Zhihua Liu, PhD^5,+^, Ajay Goel, PhD^1,14,15+^

Corresponding authors.

Email [AJGOEL@COH.ORG](mailto:AJGOEL@COH.ORG) or [liuzh@cicams.ac.cn](mailto:liuzh@cicams.ac.cn) or [xwang@surgery.cuhk.edu.hk](mailto:xwang@surgery.cuhk.edu.hk)

**SUPPLEMENTARY METHODS**

Expression profiling datasets and patient cohorts

*Public datasets:* In this study, we performed data analysis on 3 transcriptome-wide miRNA expression profiling datasets: TCGA, GSE55856, and GSE43732. The TCGA dataset consisted of tissue-based, small RNA sequencing data for 96 stage I–IV ESCC patients and 13 normal esophageal mucosae. For *in-silico* validations, two additional miRNA expression profiling datasets, GSE55856 and GSE43732, were obtained from the Gene Expression Omnibus (GEO). The GSE55856 dataset, including 108 stage II–III ESCC tissues and 108 adjacent normal esophageal tissues. The GSE43732 dataset, including 119 stage I–III ESCC tissues and 119 adjacent normal esophageal tissues. The characteristics of these three datasets are summarized in **Table S1**.

Significantly overexpressed miRNAs were first identified from each dataset using the following 3 criteria: (1) differentially expressed between ESCC and normal samples (log2 fold-change >0.5, FDR<0.05); (2) discriminative between ESCC and normal samples (AUC>0.7); and (3) upregulated in ESCC, with a relatively elevated expression to facilitate detection in serum samples (average expression > median average expression of all differentially expressed miRNAs).

*Retrospective tissue and serum cohorts:* For tissue validation of the miRNA candidates identified from public datasets, 32 tumor and 32 matched corresponding normal mucosal tissues were collected from stage I–III ESCC patients undergoing esophageal resection without any pre-operative therapy from Nagoya University Hospital, Nagoya, Japan between 2001 and 2015.

The tissue-based biomarkers were subsequently validated in large number of serum specimens from independent patient cohorts. The “*serum biomarker prioritization cohort”* included serum specimens from 50 stage I–III ESCC patients and 50 healthy controls at the Kumamoto University Hospital, Japan, enrolled between 2009 and 2011. The “*serum training cohort”* included 408 serum samples from stage I–IV ESCC patients and healthy controls collected from the Groote Schuur Hospital, Cape Town, South Africa between 2001 and 2015. The “*serum validation cohort 1”* consisted of 126 serum specimens from stage I–III ESCC patients and healthy controls collected at the Kumamoto University Hospital between 2012 and 2016. The “*serum validation cohort 2”* comprised of 165 specimens from stage I–III ESCC patients and healthy controls collected at the Nagoya University Hospital between 2001 and 2015. The Institutional Review Boards of each participating institution approved the study, and written informed consent was obtained from each participant.

The serum datasets used to evaluate the diagnostic specificity of the miRNA panel in other cancer types were obtained from GEO: GSE25609 (colorectal cancer, CRC), GSE31568 (lung cancer, LC and prostate cancer, PC) and GSE31309 (breast cancer, BC). In each dataset, the miRNA expression values were Z-normalized.

*Prospective serum cohorts:* In order to prospectively examine the circulating miRNA signature, serum specimens were collected from 178 patients with ESCC and 195 healthy individuals, matched by age and sex, who were prospectively recruited from February to July 2018 at the National Cancer Center/National Clinical Research Center for Cancer/Cancer Hospital, Chinese Academy of Medical Sciences, Beijing, China (**Table 1**). Serum specimens from ESCC patients and healthy controls were randomized into a training cohort (*Beijing-1*, 89 ESCC vs. 96 healthy) and a validation cohort (*Beijing-2*, 89 ESCC vs 99 healthy). To test the circulating miRNA signature on premalignant lesions, we also collected 27 serum specimens from prospectively recruited patients with esophagitis (n=6), low-grade intraepithelial neoplasms (n=8), and high-grade intraepithelial neoplasms (n=13) from February to July 2018 in the same hospital. Study samples were centrifuged while fresh to minimize the effects of cellular contamination and hemolysis, and stored in RNase-free tubes at −80°C. None of the patients received treatment prior to sample collection. Disease diagnoses were made by means of endoscopy, CT imaging techniques, or pathology detection. All participants provided written informed consent. This prospective cohort study was approved by the Institutional Review Board of the Cancer Hospital Chinese Academy of Medical Sciences, Beijing, China [NCC2018-97].

**
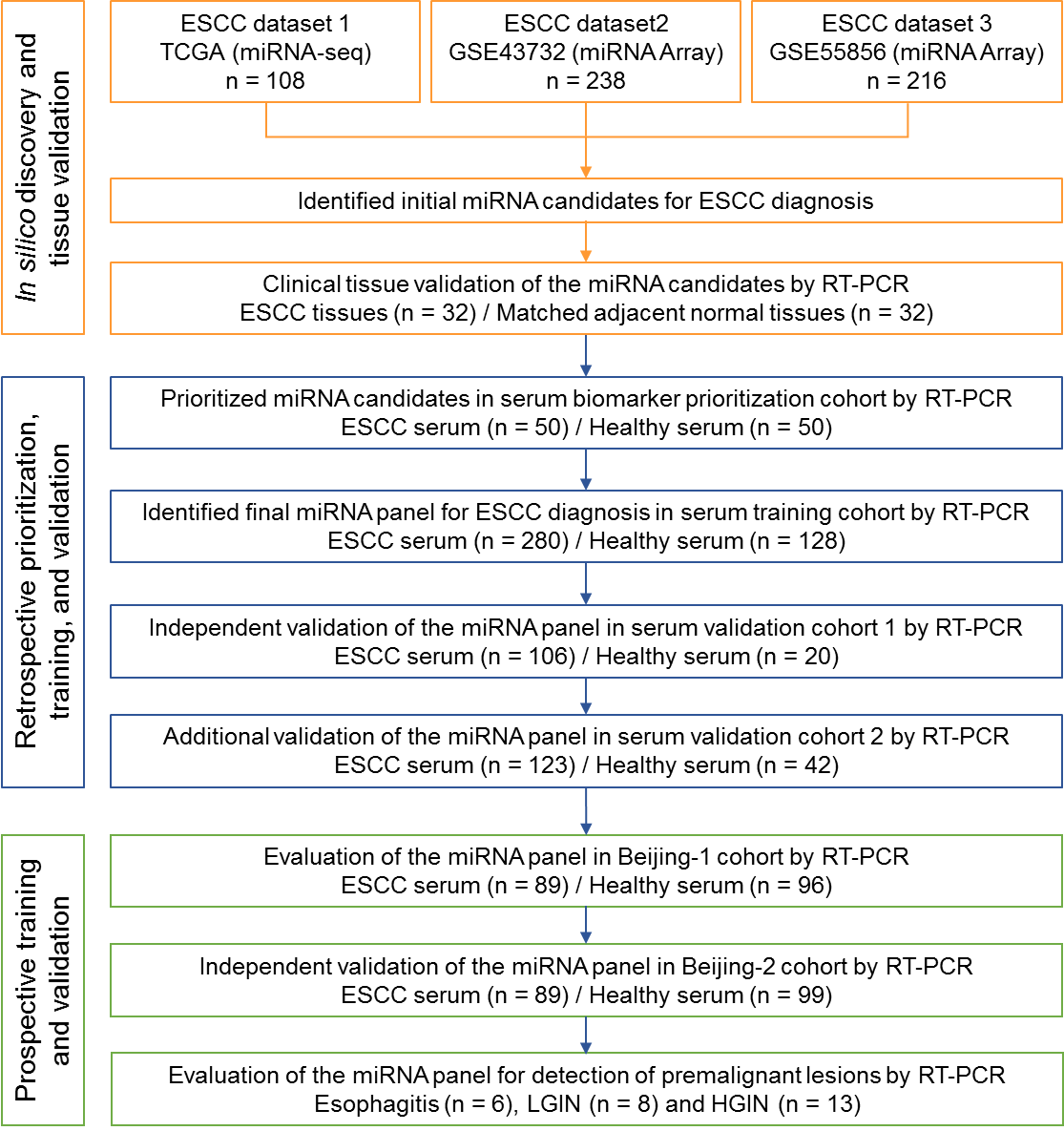
**

**Supplementary Figure 1:** Study design for the identification and validation of the circulating miRNA panel for ESCC detection.


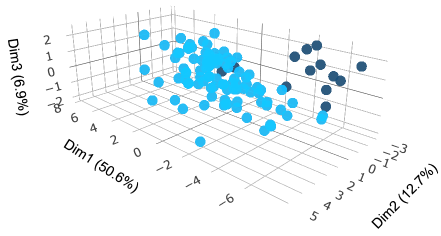

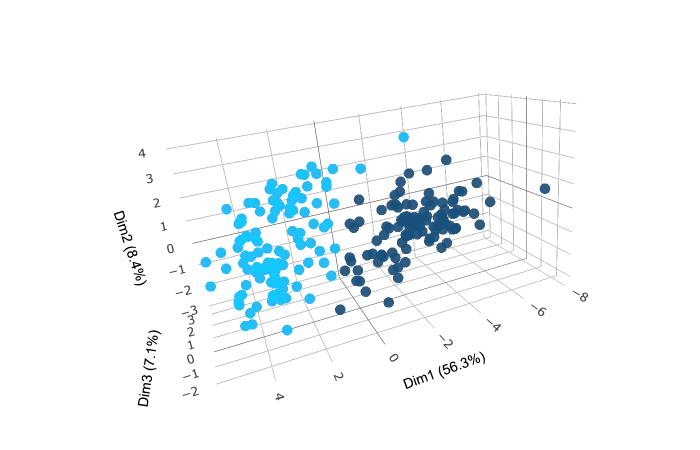

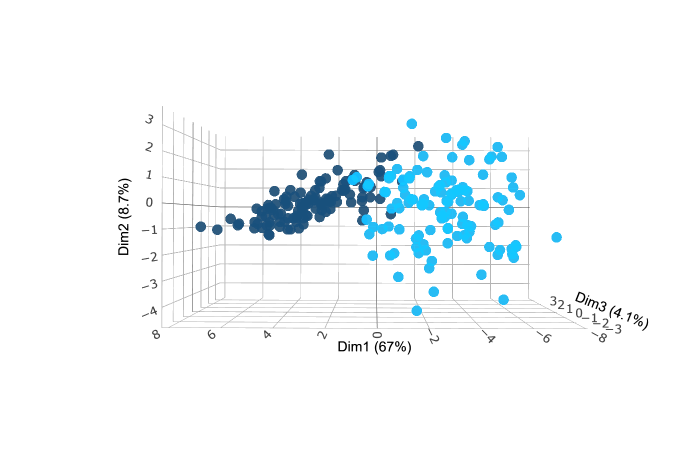


**TCGA**

**GSE55856**

**GSE43732**

**Supplementary Figure 2:** *18-miRNAs can distinguish between tumor and normal tissues.* Principal component analysis shows 18 miRNAs discriminate tumor and normal group samples.

**
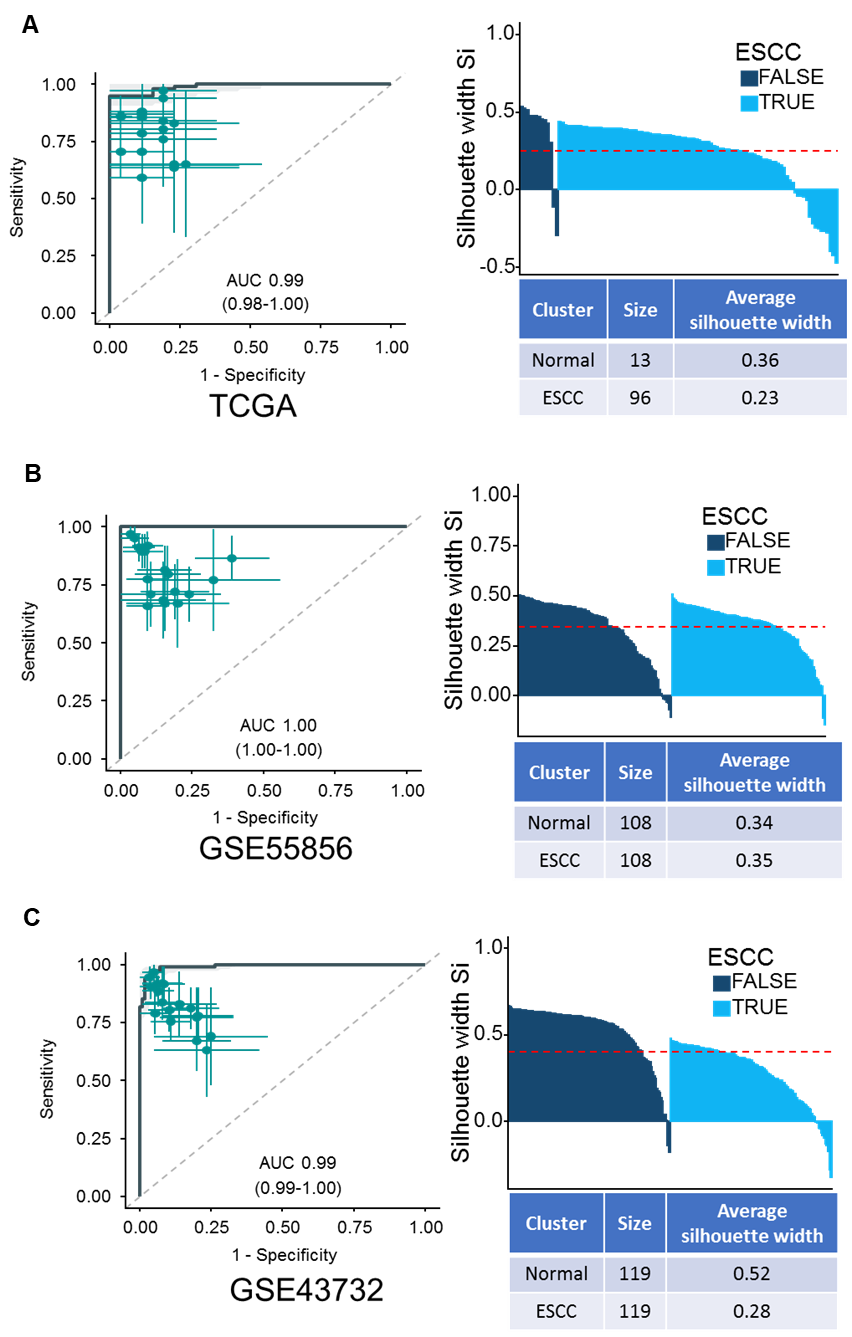
**

**Supplementary Figure 3:** *In silico* validation for 18-miRNA panel and Silhouette analysis. ROC curves (A, B and C) distinguishing ESCC tissues from normal tissues for the three miRNA expression datasets (TCGA, GSE55856, GSE43732), using a multivariate logistic regression model trained on GSE55856. A combination panel of 18 miRNAs accurately distinguished cancer tissues from normal tissues for the three datasets (AUC=0.99, 1.00, 0.99, respectively). Silhouette analysis (A, B and C) showed the stability and consistency of within ESCC and normal group (total average silhouette width 0.25, 0.35 and 0.4, respectively). Euclidean distance was calculated using 18 miRNAs.


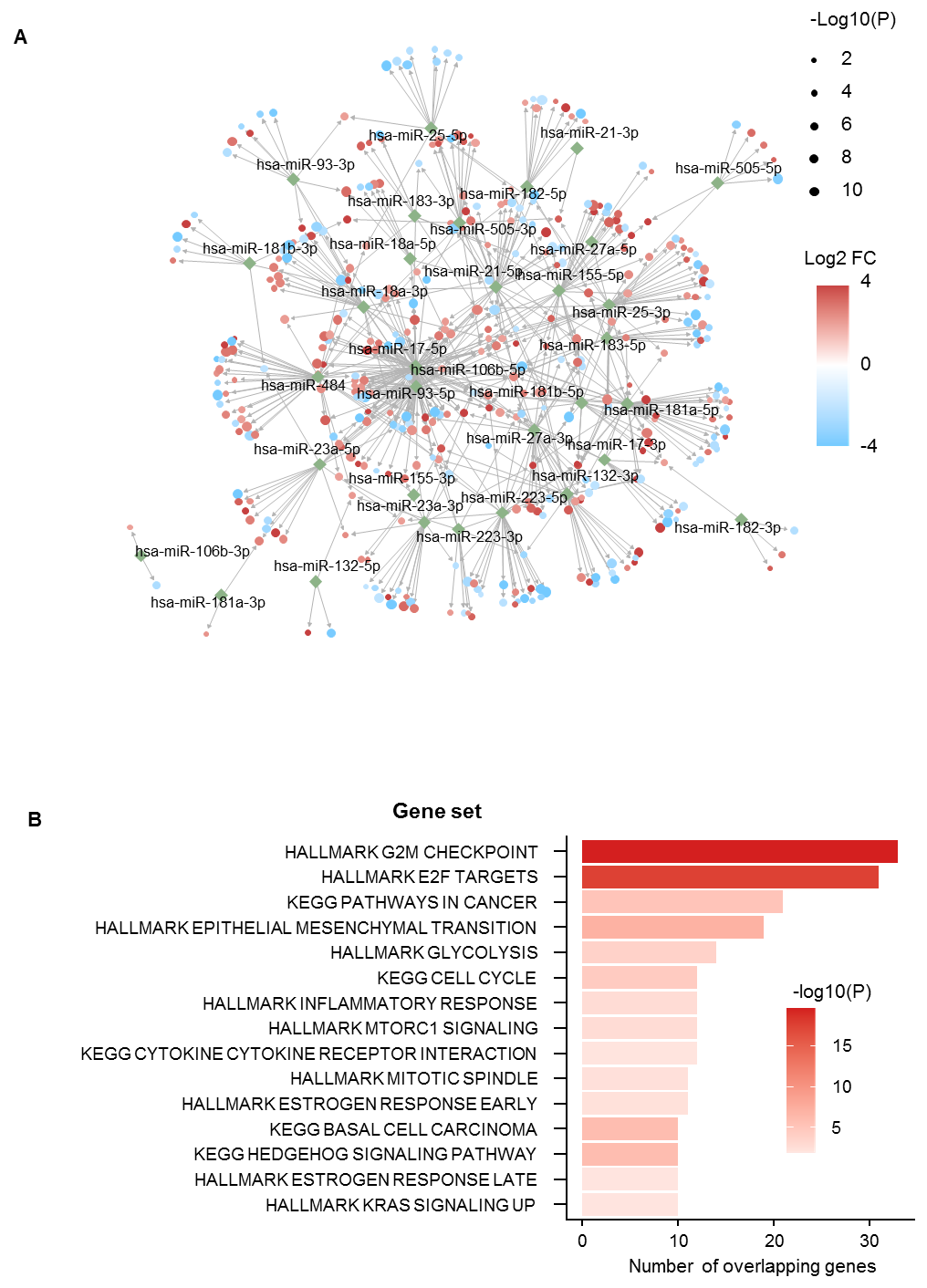


**Supplemental Figure 4:** miRNA regulatory network analysis and functional analysis of miRNA target genes. (A) Regulatory network of 18 miRNAs and 393 target mRNAs was constructed using interaction information from miRTarBase V8. Node size represents a -log10 transformed adjusted p-value, and color represents a log2-fold change between cancer and normal samples in the TCGA dataset. (B) Functional analysis using hypergeometric tests on cancer hallmark and KEGG pathways. The top 15 significantly enriched signaling pathways are illustrated in the bar plot. Bar length indicated the number of overlapping genes and color indicated the p-value.

**
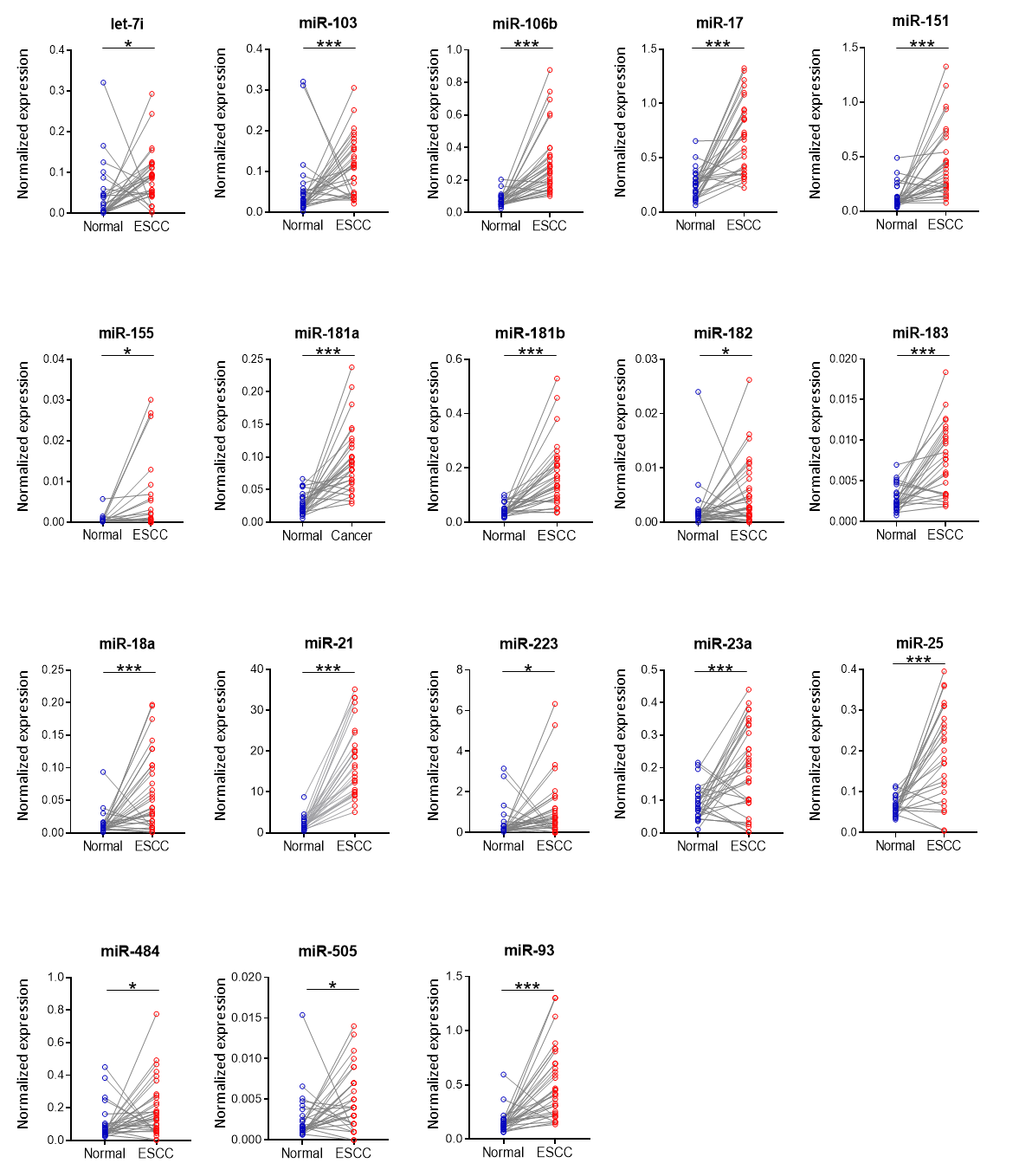
**

**Supplemental Figure 5:** Tissue validation for initial miRNA candidates. The expression of all of the 18 *in silico* miRNA candidates in ESCC tissue samples compared to adjacent normal tissues, as measured using qRT-PCR in 32 ESCC and 32 matched adjacent normal tissues. * *P* < 0.05, ** *P* < 0.01, *** *P* < 0.001

**
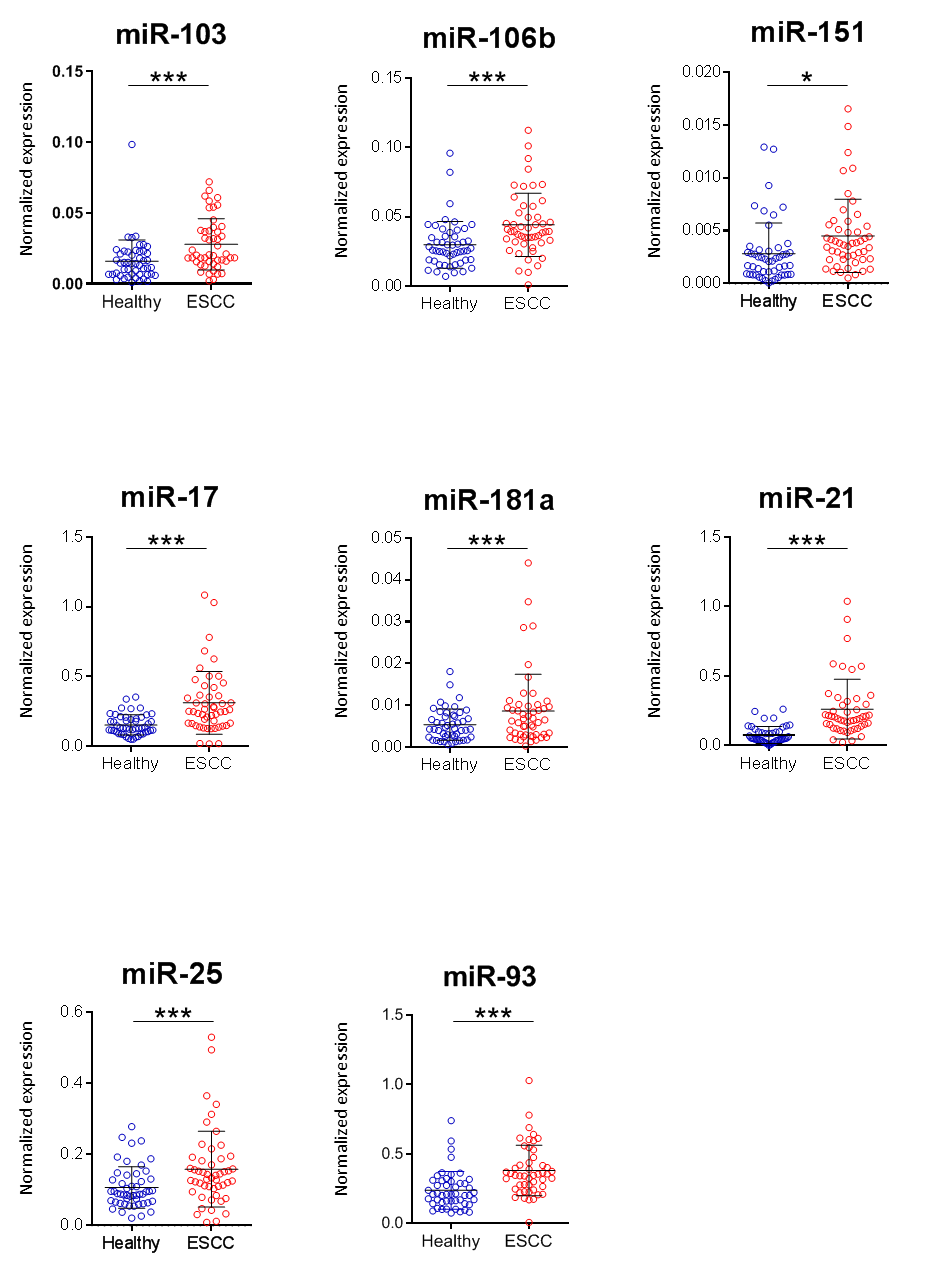
**

**Supplemental Figure 6:** Selection of circulating miRNAs in the serum biomarker prioritization cohort. Eight circulating miRNAs were significantly upregulated in serum from ESCC patients (n = 50) compared to heathy controls (n= 50) in the serum biomarker prioritization cohort. * *P* < 0.05, ** *P* < 0.01, *** *P* < 0.001


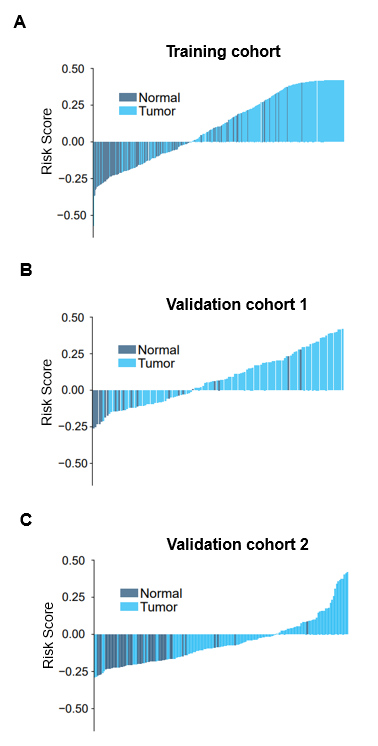


**Supplementary Figure 7***: The robustness of the miRNA-classifier in training and validation cohorts.* Waterfall plots illustrate the detailed classification of ESCC patients and healthy controls using risk scores calculated by the same risk-scoring formula and cutoff value in the retrospective cohorts including training cohort (A), validation cohort 1 (B) and Validation cohort 2 (C).


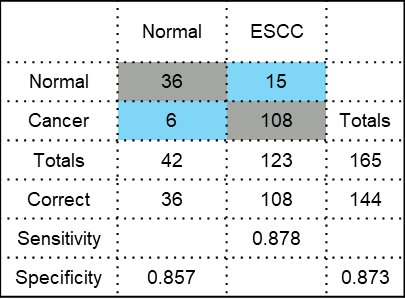


**Supplemental Figure 8:** Confusion matrices analysis for validation cohort 2. Confusion matrices were built from the same diagnostic model prediction in validation cohort 2. The 95% CI of sensitivity and specificity for eight panel members was also shown at the best threshold (calculated by Youden-Index).

**
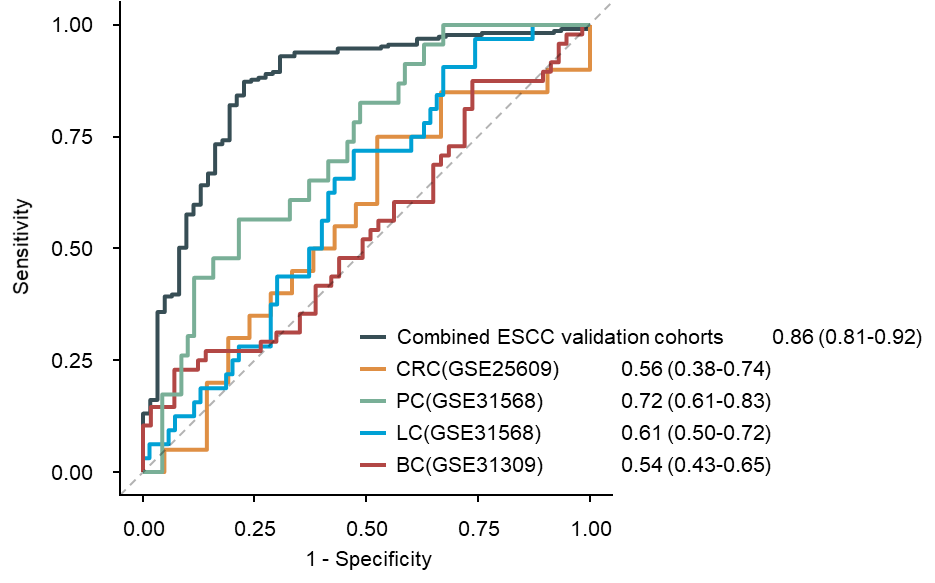
**

**Supplemental Figure 9:** Specificity analysis for the 8-miRNA panel on multiple cancer types.

ROC curves comparing the performance of the 8-miRNA panel on the combined ESCC serum validation cohorts with multiple public serum miRNA datasets for colorectal cancer (CRC), lung cancer (LC), prostate cancer (PC) and breast cancer (BC).


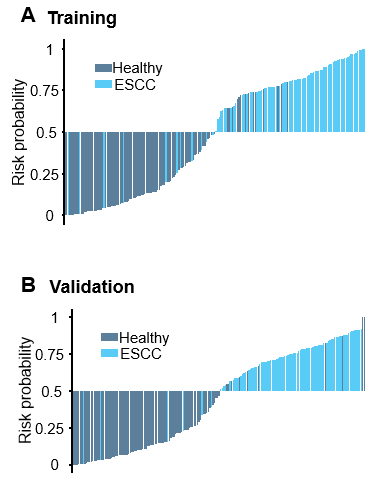


**Supplemental Figure 10**: *The robustness of the miRNA-classifier in the prospectively collected cohorts.* Waterfall plots illustrate the detailed classification of ESCC patients and healthy controls using risk scores calculated by the same risk-scoring formula and cutoff value in the prospective cohorts.

**
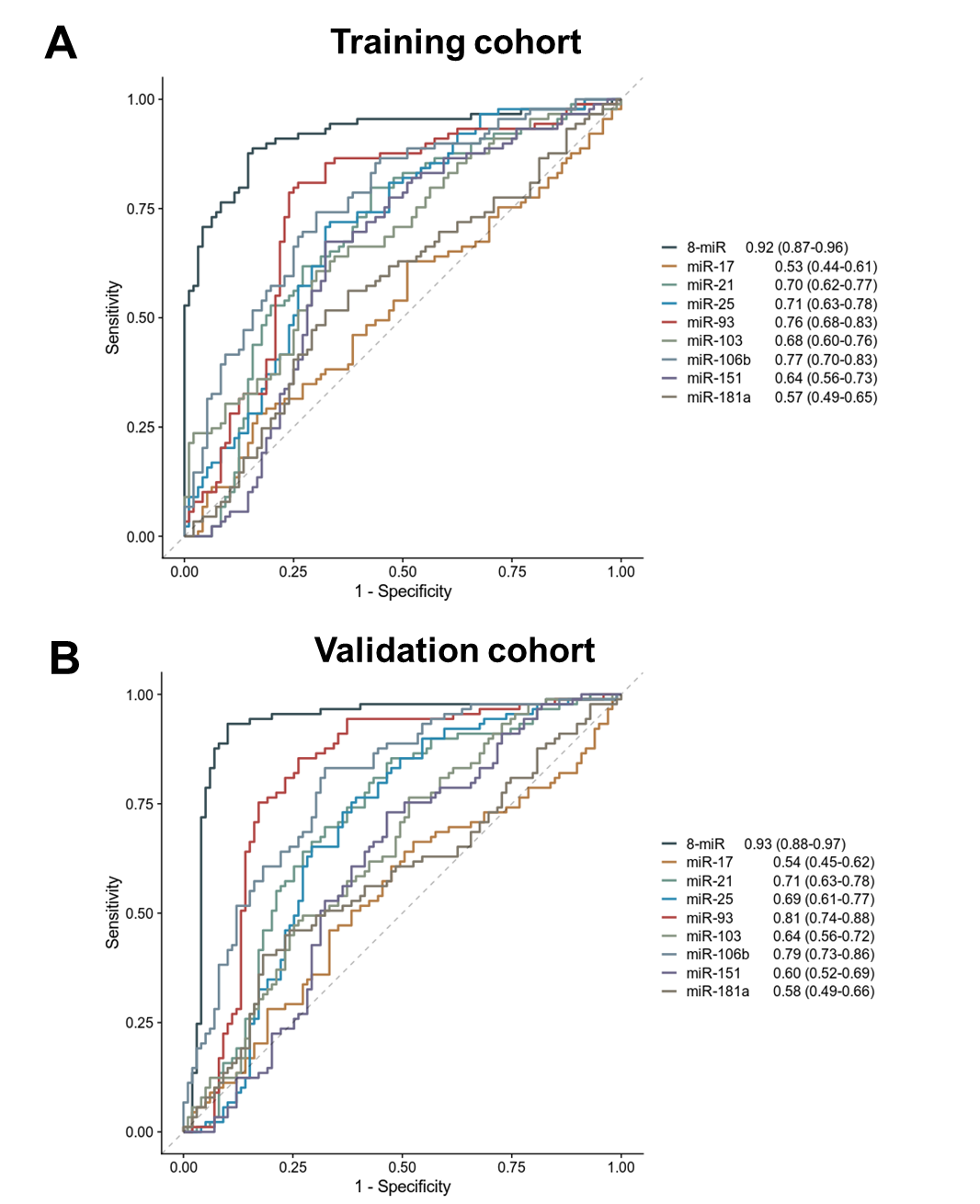
A**

**
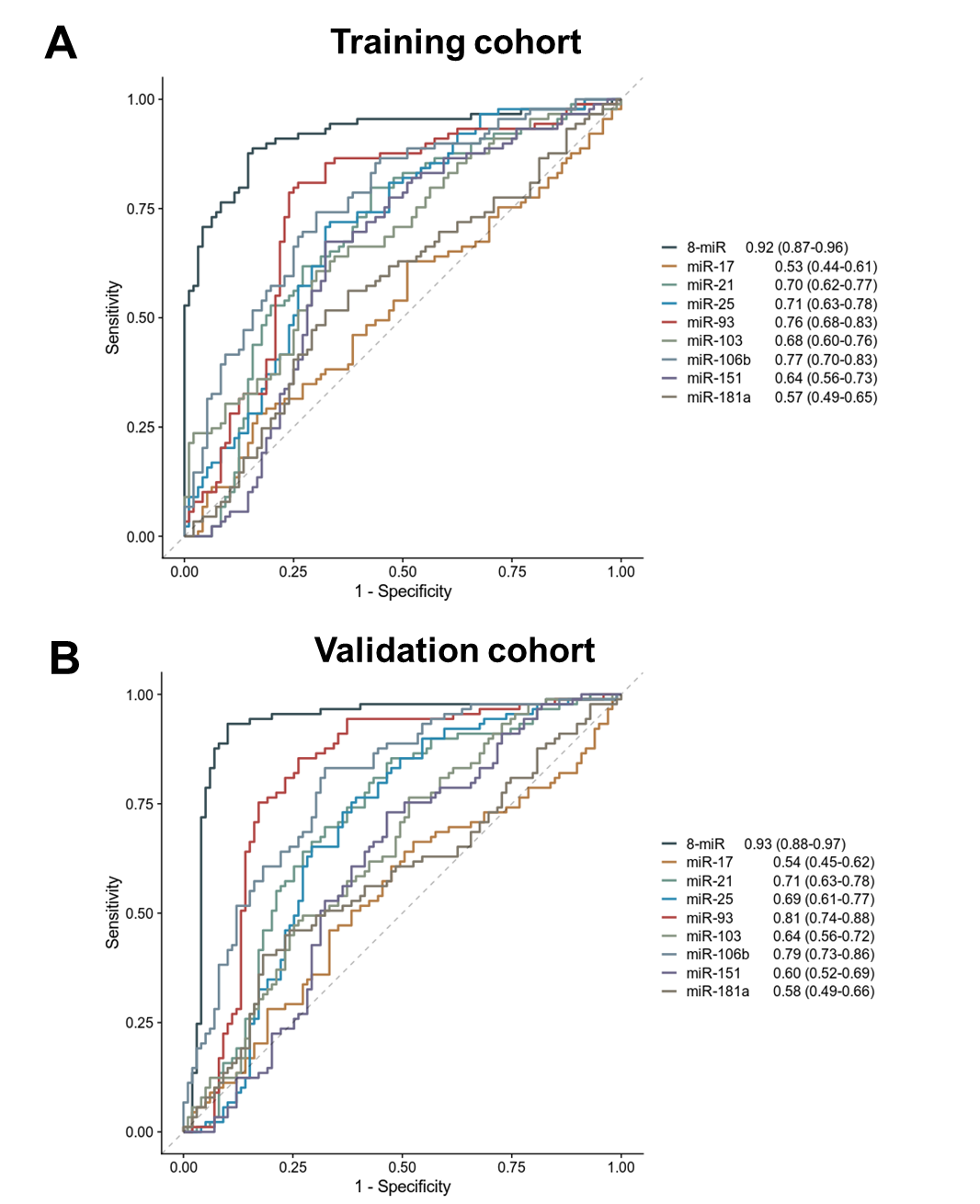
B**

**Supplemental Figure 11:** Specificity analysis for the 8-miRNA panel and individual miRNAs.

A) ROC curves comparing the performance of the 8-miRNA panel and individual miRNAs on the training cohort. B) ROC curves comparing the performance of the 8-miRNA panel and individual miRNAs on the validation cohort.

**
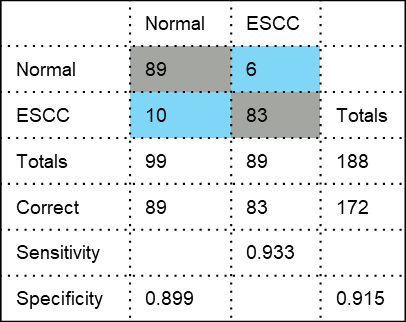
**

**Supplemental Figure 12**: Confusion matrices analysis for the Beijing-2 prospective cohort. Confusion matrices were built from the same diagnostic model prediction in Beijing-2 prospective cohort. The 95% CI of sensitivity and specificity for eight panel members was also shown at the best threshold (calculated by Youden-Index).

**SUPPLEMENTARY TABLES**

**Supplemental Table 1:** Characteristics of *in silico* discovery sets.

|  | **TCGA**  **(N = 96)** | **GSE55856**  **(N = 108)** | **GSE43732**  **(N = 119)** |
| --- | --- | --- | --- |
| **ESCC tissue** | 96 | 108 | 119 |
| Sex |  |  |  |
| Men | 81 | 102 | 98 |
| Women | 15 | 6 | 21 |
| Age (years) |  |  |  |
| Median | 57 | 66 | 59 |
| Cancer stage |  |  |  |
| I | 7 |  | 6 |
| II | 55 | 39 | 47 |
| III | 27 | 69 | 66 |
| IV | 4 |  |  |
| Unstaged | 3 |  |  |
| Race |  |  |  |
| Asian | 44 | 108 | 119 |
| Black | 6 |  |  |
| White | 43 |  |  |
| Unknown | 3 |  |  |
| **Normal Esophageal tissue** | 13 | 108^a^ | 119^a^ |

^a^ Adjacent normal esophageal tissue.

**Supplemental Table 2:** miRNA–mRNA interactions in the regulatory network.

| **miRNA** | **miRTarBase ID** | **Target Gene** | **log2FC** | **BH-adjusted *P*-value** |
| --- | --- | --- | --- | --- |
| hsa-miR-223-3p | MIRT000129 | STMN1 | 2.23 | 3.18E-08 |
| hsa-miR-21-5p | MIRT000171 | NCAPG | 2.72 | 9.02E-10 |
| hsa-miR-21-5p | MIRT000173 | BASP1 | 2.46 | 2.01E-04 |
| hsa-miR-181b-5p | MIRT000239 | GATA6 | -3.91 | 6.22E-14 |
| hsa-miR-181a-5p | MIRT000244 | GATA6 | -3.91 | 6.22E-14 |
| hsa-miR-21-5p | MIRT001121 | E2F2 | 2.04 | 6.30E-06 |
| hsa-miR-21-5p | MIRT001188 | FMOD | -2.32 | 4.79E-06 |
| hsa-miR-21-5p | MIRT001208 | TGFBI | 2.71 | 8.73E-04 |
| hsa-miR-155-5p | MIRT001491 | TRIP13 | 3.15 | 7.70E-10 |
| hsa-miR-155-5p | MIRT001496 | TACSTD2 | 3.31 | 5.25E-06 |
| hsa-miR-155-5p | MIRT001538 | METTL7A | -2.73 | 3.28E-08 |
| hsa-miR-155-5p | MIRT001541 | LY6K | 4.19 | 6.75E-05 |
| hsa-miR-155-5p | MIRT001549 | FADS1 | 2.02 | 2.83E-03 |
| hsa-miR-155-5p | MIRT001558 | CLDN1 | 3.78 | 1.79E-06 |
| hsa-miR-155-5p | MIRT001806 | JADE1 | -2.21 | 2.04E-13 |
| hsa-miR-182-5p | MIRT001992 | ADCY6 | -2.08 | 7.14E-11 |
| hsa-miR-181b-5p | MIRT003036 | VSNL1 | 4.27 | 4.27E-09 |
| hsa-miR-21-5p | MIRT003567 | SERPINB5 | 5.00 | 3.57E-08 |
| hsa-miR-93-5p | MIRT004104 | ITGB8 | 2.03 | 8.45E-06 |
| hsa-miR-155-5p | MIRT004507 | MAFB | 2.08 | 2.08E-05 |
| hsa-miR-17-5p | MIRT004710 | MUC17 | -3.83 | 3.60E-04 |
| hsa-miR-155-5p | MIRT005257 | KRT80 | 3.61 | 9.37E-05 |
| hsa-miR-21-5p | MIRT005329 | TP63 | 6.77 | 1.30E-13 |
| hsa-miR-21-5p | MIRT005330 | TGFBR3 | -2.15 | 1.91E-04 |
| hsa-miR-27a-3p | MIRT005425 | THRB | -2.39 | 4.36E-06 |
| hsa-miR-23a-3p | MIRT006280 | PPARGC1A | -2.78 | 3.23E-05 |
| hsa-miR-23a-3p | MIRT006281 | G6PC | -2.32 | 1.71E-04 |
| hsa-miR-25-3p | MIRT006454 | CCL26 | 2.97 | 3.31E-04 |
| hsa-miR-27a-3p | MIRT006557 | MMP13 | 6.54 | 5.44E-05 |
| hsa-miR-223-3p | MIRT006559 | SLC2A4 | -2.66 | 1.19E-04 |
| hsa-miR-223-3p | MIRT006682 | ARTN | 3.36 | 6.05E-05 |
| hsa-miR-182-5p | MIRT006823 | FGF9 | -2.07 | 2.93E-03 |
| hsa-miR-182-5p | MIRT007226 | SNAI2 | 3.27 | 1.67E-09 |
| hsa-miR-106b-5p | MIRT020419 | LINC00483 | -2.86 | 5.97E-07 |
| hsa-miR-106b-5p | MIRT020421 | SYT4 | -4.31 | 6.66E-08 |
| hsa-miR-106b-5p | MIRT020426 | KIF23 | 2.71 | 2.19E-11 |
| hsa-miR-106b-5p | MIRT020433 | SBSN | 6.34 | 1.29E-03 |
| hsa-miR-106b-5p | MIRT020449 | HMGB3 | 2.41 | 2.90E-08 |
| hsa-miR-155-5p | MIRT020525 | CAB39L | -2.24 | 2.10E-09 |
| hsa-miR-155-5p | MIRT020585 | SCD | 2.02 | 1.70E-04 |
| hsa-miR-155-5p | MIRT020589 | AURKA | 2.09 | 4.29E-09 |
| hsa-miR-155-5p | MIRT020611 | CEP55 | 3.08 | 9.33E-10 |
| hsa-miR-155-5p | MIRT020615 | PLK1 | 2.48 | 2.14E-08 |
| hsa-miR-155-5p | MIRT020616 | AURKB | 3.34 | 3.77E-11 |
| hsa-miR-155-5p | MIRT020622 | RRM2 | 2.93 | 6.73E-09 |
| hsa-miR-155-5p | MIRT020647 | KRT6B | 7.70 | 4.22E-05 |
| hsa-miR-155-5p | MIRT020693 | NCAPG | 2.72 | 9.02E-10 |
| hsa-miR-155-5p | MIRT020701 | CD109 | 3.19 | 1.15E-08 |
| hsa-miR-155-5p | MIRT020773 | OLR1 | 3.26 | 1.71E-04 |
| hsa-miR-155-5p | MIRT020784 | ZNF493 | -2.35 | 2.04E-06 |
| hsa-miR-155-5p | MIRT020790 | JCHAIN | -3.40 | 3.63E-06 |
| hsa-miR-155-5p | MIRT020965 | WNT5A | 2.39 | 8.54E-04 |
| hsa-miR-155-5p | MIRT020979 | CXCL8 | 4.02 | 3.14E-05 |
| hsa-miR-155-5p | MIRT021007 | CDH13 | 2.71 | 5.36E-05 |
| hsa-miR-155-5p | MIRT021033 | PYGL | 2.99 | 1.76E-04 |
| hsa-miR-155-5p | MIRT021036 | KIF14 | 3.12 | 4.96E-11 |
| hsa-miR-155-5p | MIRT021061 | GATM | -2.61 | 5.21E-07 |
| hsa-miR-155-5p | MIRT021062 | E2F2 | 2.04 | 6.30E-06 |
| hsa-miR-132-3p | MIRT021719 | CDH3 | 4.52 | 7.09E-10 |
| hsa-miR-132-3p | MIRT021725 | WNT3A | 4.80 | 6.81E-06 |
| hsa-miR-132-3p | MIRT021736 | MUC17 | -3.83 | 3.60E-04 |
| hsa-miR-132-3p | MIRT021737 | CA1 | -2.96 | 2.52E-07 |
| hsa-miR-132-3p | MIRT021744 | STMN1 | 2.23 | 3.18E-08 |
| hsa-miR-132-3p | MIRT021747 | TRIM36 | -2.44 | 1.48E-07 |
| hsa-miR-132-3p | MIRT021750 | GCNT1 | -2.35 | 9.83E-08 |
| hsa-miR-132-3p | MIRT021754 | SYT14 | 2.61 | 3.02E-03 |
| hsa-miR-132-3p | MIRT021778 | KRT6C | 6.40 | 1.20E-03 |
| hsa-miR-132-3p | MIRT021796 | MMP13 | 6.54 | 5.44E-05 |
| hsa-miR-132-3p | MIRT021799 | RNF128 | -2.48 | 6.06E-04 |
| hsa-miR-132-3p | MIRT021807 | B3GAT1 | -4.63 | 1.56E-09 |
| hsa-miR-132-3p | MIRT021814 | ADAMTSL1 | -2.25 | 3.45E-04 |
| hsa-miR-132-3p | MIRT021816 | CFTR | -3.04 | 9.36E-05 |
| hsa-miR-132-3p | MIRT021820 | ADGRF4 | 4.54 | 5.44E-05 |
| hsa-miR-132-3p | MIRT021839 | ECT2 | 2.38 | 3.79E-08 |
| hsa-miR-132-3p | MIRT021860 | MUC13 | -5.19 | 1.14E-09 |
| hsa-miR-132-3p | MIRT021865 | CCNA2 | 2.52 | 2.36E-09 |
| hsa-miR-132-3p | MIRT021866 | CCNB1 | 2.69 | 6.89E-11 |
| hsa-miR-183-5p | MIRT025014 | TXNIP | -2.06 | 1.05E-05 |
| hsa-miR-183-5p | MIRT025027 | BCL11B | 2.83 | 2.08E-05 |
| hsa-miR-181a-5p | MIRT025040 | CHRFAM7A | -2.45 | 3.04E-06 |
| hsa-miR-181a-5p | MIRT025042 | RASSF6 | -2.40 | 9.73E-06 |
| hsa-miR-181a-5p | MIRT025061 | WNT3A | 4.80 | 6.81E-06 |
| hsa-miR-181a-5p | MIRT025064 | TMEM45A | 2.93 | 1.01E-04 |
| hsa-miR-181a-5p | MIRT025069 | NMRK2 | -2.12 | 1.13E-03 |
| hsa-miR-181a-5p | MIRT025070 | WNT2 | 3.10 | 3.47E-04 |
| hsa-miR-181a-5p | MIRT025075 | GCNT1 | -2.35 | 9.83E-08 |
| hsa-miR-181a-5p | MIRT025082 | NOL4 | -2.32 | 1.23E-03 |
| hsa-miR-181a-5p | MIRT025105 | SCD | 2.02 | 1.70E-04 |
| hsa-miR-181a-5p | MIRT025118 | TGFBR3 | -2.15 | 1.91E-04 |
| hsa-miR-181a-5p | MIRT025134 | OFCC1 | 2.89 | 3.64E-03 |
| hsa-miR-181a-5p | MIRT025136 | NKX3-2 | 2.90 | 1.24E-04 |
| hsa-miR-181a-5p | MIRT025137 | OTX2 | 2.03 | 1.63E-03 |
| hsa-miR-181a-5p | MIRT025144 | EPHA5 | -3.16 | 1.02E-04 |
| hsa-miR-181a-5p | MIRT025163 | PCLAF | 2.57 | 4.58E-09 |
| hsa-miR-181a-5p | MIRT025177 | TMPRSS11A | 4.68 | 4.31E-03 |
| hsa-miR-181a-5p | MIRT025178 | SNAI2 | 3.27 | 1.67E-09 |
| hsa-miR-181a-5p | MIRT025183 | PRLR | -2.93 | 2.64E-05 |
| hsa-miR-181a-5p | MIRT025194 | ATP8A1 | -3.62 | 8.05E-12 |
| hsa-miR-93-5p | MIRT027944 | TMEM100 | -3.70 | 5.79E-11 |
| hsa-miR-93-5p | MIRT027950 | ZIC5 | 4.44 | 3.89E-04 |
| hsa-miR-93-5p | MIRT027999 | SYBU | -3.52 | 7.97E-08 |
| hsa-miR-93-5p | MIRT028012 | RUNX3 | 2.36 | 5.32E-06 |
| hsa-miR-93-5p | MIRT028041 | STC2 | 3.44 | 1.10E-05 |
| hsa-miR-93-5p | MIRT028167 | PRUNE2 | -2.56 | 2.70E-03 |
| hsa-miR-27a-3p | MIRT028718 | ECT2 | 2.38 | 3.79E-08 |
| hsa-miR-27a-3p | MIRT028721 | HOXC6 | 3.61 | 5.23E-08 |
| hsa-miR-27a-3p | MIRT028730 | ARL4C | 2.18 | 3.01E-06 |
| hsa-miR-27a-3p | MIRT028732 | KCTD14 | -2.41 | 4.57E-05 |
| hsa-miR-27a-3p | MIRT028740 | LIFR | -3.04 | 1.01E-07 |
| hsa-miR-21-5p | MIRT030666 | COL5A2 | 2.28 | 1.16E-03 |
| hsa-miR-21-5p | MIRT030689 | MGAT4A | -2.07 | 5.62E-08 |
| hsa-miR-21-5p | MIRT030730 | TGFB1 | 2.19 | 5.73E-07 |
| hsa-miR-21-5p | MIRT030746 | PDGFD | -2.65 | 2.65E-08 |
| hsa-miR-21-5p | MIRT030748 | SAMD5 | -2.45 | 4.02E-04 |
| hsa-miR-21-5p | MIRT030770 | JADE1 | -2.21 | 2.04E-13 |
| hsa-miR-21-5p | MIRT030776 | LONRF2 | -3.70 | 7.19E-06 |
| hsa-miR-21-5p | MIRT030810 | MUC1 | -3.50 | 4.88E-12 |
| hsa-miR-21-5p | MIRT030815 | KNL1 | 2.32 | 4.04E-07 |
| hsa-miR-21-5p | MIRT030838 | DDAH1 | -2.69 | 3.65E-10 |
| hsa-miR-21-5p | MIRT030857 | LIFR | -3.04 | 1.01E-07 |
| hsa-miR-21-5p | MIRT030871 | MMP9 | 3.33 | 2.12E-04 |
| hsa-miR-21-5p | MIRT030881 | PPFIA4 | 2.45 | 3.72E-04 |
| hsa-miR-21-5p | MIRT030900 | TMEM56 | -2.33 | 5.02E-05 |
| hsa-miR-21-5p | MIRT030914 | TOP2A | 2.91 | 2.53E-09 |
| hsa-miR-21-5p | MIRT030942 | ADGRG2 | -2.57 | 2.49E-07 |
| hsa-miR-21-5p | MIRT030983 | WNT5A | 2.39 | 8.54E-04 |
| hsa-miR-21-5p | MIRT031012 | OLR1 | 3.26 | 1.71E-04 |
| hsa-miR-21-5p | MIRT031039 | B3GNT5 | 2.16 | 3.92E-06 |
| hsa-miR-21-5p | MIRT031042 | HMGB3 | 2.41 | 2.90E-08 |
| hsa-miR-21-5p | MIRT031045 | NETO2 | 2.11 | 1.42E-05 |
| hsa-miR-106b-3p | MIRT038560 | ADAMTS2 | 2.03 | 4.10E-03 |
| hsa-miR-93-3p | MIRT038720 | HASPIN | 2.08 | 5.77E-07 |
| hsa-miR-93-3p | MIRT038755 | KIAA1549L | 3.87 | 9.31E-05 |
| hsa-miR-93-3p | MIRT038774 | CENPF | 2.97 | 1.12E-09 |
| hsa-miR-93-3p | MIRT038799 | HMMR | 2.50 | 6.25E-08 |
| hsa-miR-93-3p | MIRT038870 | CDC20 | 3.14 | 9.54E-11 |
| hsa-miR-18a-3p | MIRT040758 | GINS1 | 2.59 | 6.58E-10 |
| hsa-miR-18a-3p | MIRT040765 | C6orf222 | -5.35 | 5.52E-13 |
| hsa-miR-18a-3p | MIRT040773 | DPF1 | 2.77 | 3.08E-05 |
| hsa-miR-18a-3p | MIRT040781 | HIST2H2AA3 | 2.04 | 4.09E-04 |
| hsa-miR-18a-3p | MIRT040791 | SEMA4G | -2.42 | 1.63E-09 |
| hsa-miR-18a-3p | MIRT040802 | FADS2 | 2.29 | 1.60E-03 |
| hsa-miR-18a-3p | MIRT040814 | ADAD2 | 2.33 | 9.36E-04 |
| hsa-miR-18a-3p | MIRT040846 | FGF11 | 2.71 | 2.81E-04 |
| hsa-miR-18a-3p | MIRT040857 | CDCA2 | 2.77 | 6.28E-09 |
| hsa-miR-18a-3p | MIRT040900 | DDAH1 | -2.69 | 3.65E-10 |
| hsa-miR-18a-3p | MIRT040934 | ASF1B | 2.64 | 2.38E-08 |
| hsa-miR-18a-3p | MIRT040951 | SRL | -2.25 | 2.70E-06 |
| hsa-miR-18a-3p | MIRT040967 | PMAIP1 | 2.60 | 1.81E-09 |
| hsa-miR-18a-3p | MIRT040968 | TYMS | 2.13 | 5.50E-08 |
| hsa-miR-505-3p | MIRT040993 | PRC1 | 2.28 | 2.08E-09 |
| hsa-miR-505-3p | MIRT041009 | TUBB3 | 2.36 | 4.29E-04 |
| hsa-miR-505-3p | MIRT041019 | MYBL2 | 3.14 | 8.21E-10 |
| hsa-miR-505-3p | MIRT041044 | MKI67 | 2.48 | 1.63E-07 |
| hsa-miR-484 | MIRT041674 | TBX1 | 2.57 | 1.46E-03 |
| hsa-miR-484 | MIRT041721 | SLC43A1 | -2.95 | 2.09E-11 |
| hsa-miR-484 | MIRT041728 | BIRC5 | 3.40 | 2.08E-12 |
| hsa-miR-484 | MIRT041751 | NCAPH | 3.03 | 1.76E-10 |
| hsa-miR-484 | MIRT041773 | AUNIP | 2.05 | 9.83E-08 |
| hsa-miR-484 | MIRT041808 | SLC2A1 | 3.58 | 7.69E-07 |
| hsa-miR-484 | MIRT041847 | SERPINH1 | 2.05 | 2.08E-05 |
| hsa-miR-484 | MIRT041876 | MKI67 | 2.48 | 1.63E-07 |
| hsa-miR-484 | MIRT041879 | ORC6 | 2.51 | 2.18E-11 |
| hsa-miR-484 | MIRT041954 | CYSTM1 | -3.66 | 4.44E-17 |
| hsa-miR-484 | MIRT041961 | SCD | 2.02 | 1.70E-04 |
| hsa-miR-484 | MIRT041980 | RRM2 | 2.93 | 6.73E-09 |
| hsa-miR-484 | MIRT041983 | ADRA1A | -2.51 | 1.90E-04 |
| hsa-miR-484 | MIRT041985 | SPHK1 | 2.67 | 1.89E-07 |
| hsa-miR-484 | MIRT041986 | APLP1 | -2.69 | 2.08E-05 |
| hsa-miR-484 | MIRT042085 | CLDN7 | 2.51 | 4.11E-04 |
| hsa-miR-484 | MIRT042105 | GRIA4 | -4.87 | 1.44E-11 |
| hsa-miR-484 | MIRT042117 | HIST1H3H | 2.37 | 4.31E-04 |
| hsa-miR-484 | MIRT042126 | UBE2S | 2.47 | 4.93E-10 |
| hsa-miR-484 | MIRT042139 | CNFN | 4.09 | 9.65E-03 |
| hsa-miR-484 | MIRT042161 | JAG2 | 2.14 | 2.08E-05 |
| hsa-miR-484 | MIRT042177 | STMN1 | 2.23 | 3.18E-08 |
| hsa-miR-484 | MIRT042194 | NOTCH3 | 2.73 | 5.88E-07 |
| hsa-miR-484 | MIRT042226 | TROAP | 2.95 | 2.11E-11 |
| hsa-miR-484 | MIRT042270 | LMNB1 | 2.05 | 6.26E-08 |
| hsa-miR-484 | MIRT042289 | TYMS | 2.13 | 5.50E-08 |
| hsa-miR-484 | MIRT042290 | GTSE1 | 2.56 | 1.46E-08 |
| hsa-miR-484 | MIRT042340 | HOXC11 | 3.99 | 1.01E-05 |
| hsa-miR-484 | MIRT042342 | LONRF2 | -3.70 | 7.19E-06 |
| hsa-miR-106b-5p | MIRT044251 | DGKD | -2.77 | 2.84E-15 |
| hsa-miR-106b-5p | MIRT044255 | CENPM | 2.36 | 4.31E-08 |
| hsa-miR-106b-5p | MIRT044354 | FAM171A2 | 2.54 | 2.70E-06 |
| hsa-miR-106b-5p | MIRT044370 | HMMR | 2.50 | 6.25E-08 |
| hsa-miR-183-5p | MIRT047056 | GATA6 | -3.91 | 6.22E-14 |
| hsa-miR-183-5p | MIRT047127 | CCNB1 | 2.69 | 6.89E-11 |
| hsa-miR-183-5p | MIRT047135 | FAT2 | 5.97 | 7.12E-10 |
| hsa-miR-183-5p | MIRT047147 | MCM4 | 2.31 | 5.90E-10 |
| hsa-miR-183-5p | MIRT047150 | ITGA8 | -2.92 | 5.48E-07 |
| hsa-miR-182-5p | MIRT047176 | BRIP1 | 2.15 | 5.72E-08 |
| hsa-miR-181b-5p | MIRT047247 | KPNA2 | 2.15 | 1.09E-08 |
| hsa-miR-181b-5p | MIRT047253 | HIST1H1E | 2.29 | 4.72E-06 |
| hsa-miR-181a-5p | MIRT047338 | PPP1R9A | -4.31 | 9.21E-09 |
| hsa-miR-93-5p | MIRT048739 | ZIC2 | 3.95 | 7.78E-04 |
| hsa-miR-93-5p | MIRT048751 | KPNA2 | 2.15 | 1.09E-08 |
| hsa-miR-93-5p | MIRT048768 | CDHR1 | 3.74 | 9.70E-04 |
| hsa-miR-93-5p | MIRT048777 | DNMT3B | 2.45 | 3.54E-07 |
| hsa-miR-93-5p | MIRT048806 | BIRC5 | 3.40 | 2.08E-12 |
| hsa-miR-93-5p | MIRT048813 | HIST1H3B | 3.21 | 1.80E-07 |
| hsa-miR-93-5p | MIRT048838 | MAD2L1 | 2.50 | 3.11E-10 |
| hsa-miR-93-5p | MIRT048878 | PYGL | 2.99 | 1.76E-04 |
| hsa-miR-93-5p | MIRT048909 | GLI3 | 2.31 | 5.82E-06 |
| hsa-miR-27a-3p | MIRT050017 | CEP55 | 3.08 | 9.33E-10 |
| hsa-miR-27a-3p | MIRT050033 | ATP7B | -2.10 | 1.52E-08 |
| hsa-miR-25-3p | MIRT050223 | RNASEH2A | 2.16 | 8.19E-09 |
| hsa-miR-25-3p | MIRT050230 | CTSE | -9.34 | 1.57E-17 |
| hsa-miR-25-3p | MIRT050233 | HYAL1 | -2.03 | 2.30E-04 |
| hsa-miR-25-3p | MIRT050240 | TUSC3 | 2.28 | 3.47E-04 |
| hsa-miR-25-3p | MIRT050242 | HIST2H2AA3 | 2.04 | 4.09E-04 |
| hsa-miR-25-3p | MIRT050243 | ADAMTSL1 | -2.25 | 3.45E-04 |
| hsa-miR-25-3p | MIRT050282 | SYNPR | -3.74 | 1.59E-09 |
| hsa-miR-25-3p | MIRT050314 | CLSPN | 2.44 | 1.27E-09 |
| hsa-miR-25-3p | MIRT050316 | RYR2 | -2.18 | 1.38E-03 |
| hsa-miR-25-3p | MIRT050321 | KIF23 | 2.71 | 2.19E-11 |
| hsa-miR-25-3p | MIRT050335 | PKDCC | -2.09 | 1.57E-03 |
| hsa-miR-23a-3p | MIRT050411 | KIF20A | 3.22 | 3.26E-11 |
| hsa-miR-23a-3p | MIRT050425 | HIST1H3B | 3.21 | 1.80E-07 |
| hsa-miR-18a-5p | MIRT050653 | DDAH1 | -2.69 | 3.65E-10 |
| hsa-miR-18a-5p | MIRT050708 | CDC20 | 3.14 | 9.54E-11 |
| hsa-miR-18a-5p | MIRT050725 | PKMYT1 | 3.39 | 1.66E-11 |
| hsa-miR-18a-5p | MIRT050732 | CDCA5 | 3.06 | 1.50E-10 |
| hsa-miR-17-3p | MIRT050744 | KPNA2 | 2.15 | 1.09E-08 |
| hsa-miR-17-3p | MIRT050745 | FSCN1 | 4.19 | 3.99E-11 |
| hsa-miR-17-3p | MIRT050749 | FAT2 | 5.97 | 7.12E-10 |
| hsa-miR-17-5p | MIRT050827 | HIST1H2AM | 2.63 | 2.11E-06 |
| hsa-miR-17-5p | MIRT050848 | UBE2S | 2.47 | 4.93E-10 |
| hsa-miR-17-5p | MIRT050849 | RYR2 | -2.18 | 1.38E-03 |
| hsa-miR-17-5p | MIRT050850 | HIST2H2AA3 | 2.04 | 4.09E-04 |
| hsa-miR-17-5p | MIRT050874 | UBE2C | 3.15 | 6.89E-11 |
| hsa-miR-17-5p | MIRT050884 | DEPDC1 | 3.00 | 1.31E-10 |
| hsa-miR-17-5p | MIRT050966 | FANCA | 2.05 | 4.96E-09 |
| hsa-miR-17-5p | MIRT050987 | PTTG1 | 3.14 | 4.98E-12 |
| hsa-miR-17-5p | MIRT051025 | STIL | 2.04 | 3.77E-08 |
| hsa-miR-17-5p | MIRT051037 | MKI67 | 2.48 | 1.63E-07 |
| hsa-miR-93-5p | MIRT053085 | SLC2A4 | -2.66 | 1.19E-04 |
| hsa-miR-223-3p | MIRT053107 | ECT2 | 2.38 | 3.79E-08 |
| hsa-miR-17-5p | MIRT053108 | ITGB8 | 2.03 | 8.45E-06 |
| hsa-miR-21-5p | MIRT053173 | HPGD | -4.09 | 9.00E-11 |
| hsa-miR-181a-5p | MIRT053666 | PCDHAC2 | -2.60 | 1.67E-04 |
| hsa-miR-181a-5p | MIRT053689 | CDH13 | 2.71 | 5.36E-05 |
| hsa-miR-181a-5p | MIRT053694 | MMP14 | 2.02 | 3.10E-06 |
| hsa-miR-106b-5p | MIRT054405 | TWIST1 | 2.84 | 2.08E-05 |
| hsa-miR-23a-3p | MIRT054472 | CXCL8 | 4.02 | 3.14E-05 |
| hsa-miR-27a-3p | MIRT054499 | PPARG | -2.83 | 3.19E-07 |
| hsa-miR-155-5p | MIRT054639 | RAD51 | 2.49 | 3.57E-09 |
| hsa-miR-183-5p | MIRT054655 | SNAI2 | 3.27 | 1.67E-09 |
| hsa-miR-223-3p | MIRT054680 | TOX | -2.05 | 1.44E-04 |
| hsa-miR-27a-3p | MIRT054696 | HOXA10 | 2.80 | 1.64E-04 |
| hsa-miR-155-5p | MIRT054752 | THRB | -2.39 | 4.36E-06 |
| hsa-miR-181b-5p | MIRT055114 | SCD | 2.02 | 1.70E-04 |
| hsa-miR-17-5p | MIRT058911 | FAM46C | -2.82 | 4.03E-08 |
| hsa-miR-93-5p | MIRT058913 | FAM46C | -2.82 | 4.03E-08 |
| hsa-miR-106b-5p | MIRT058915 | FAM46C | -2.82 | 4.03E-08 |
| hsa-miR-27a-3p | MIRT059144 | TXNIP | -2.06 | 1.05E-05 |
| hsa-miR-25-3p | MIRT066473 | HMGA2 | 3.34 | 2.63E-03 |
| hsa-miR-27a-3p | MIRT076088 | ADORA2B | 3.35 | 1.14E-10 |
| hsa-miR-17-5p | MIRT084343 | RRM2 | 2.93 | 6.73E-09 |
| hsa-miR-93-5p | MIRT084345 | RRM2 | 2.93 | 6.73E-09 |
| hsa-miR-106b-5p | MIRT084347 | RRM2 | 2.93 | 6.73E-09 |
| hsa-miR-17-5p | MIRT100276 | MICB | 2.33 | 1.03E-04 |
| hsa-miR-93-5p | MIRT100278 | MICB | 2.33 | 1.03E-04 |
| hsa-miR-106b-5p | MIRT100281 | MICB | 2.33 | 1.03E-04 |
| hsa-miR-181b-3p | MIRT118893 | LONRF2 | -3.70 | 7.19E-06 |
| hsa-miR-17-5p | MIRT124124 | GINS4 | 2.38 | 2.20E-06 |
| hsa-miR-93-5p | MIRT124126 | GINS4 | 2.38 | 2.20E-06 |
| hsa-miR-106b-5p | MIRT124131 | GINS4 | 2.38 | 2.20E-06 |
| hsa-miR-17-5p | MIRT130067 | TXNIP | -2.06 | 1.05E-05 |
| hsa-miR-93-5p | MIRT130073 | TXNIP | -2.06 | 1.05E-05 |
| hsa-miR-106b-5p | MIRT130091 | TXNIP | -2.06 | 1.05E-05 |
| hsa-miR-17-5p | MIRT138345 | FRMD6 | 2.69 | 3.07E-08 |
| hsa-miR-93-5p | MIRT138351 | FRMD6 | 2.69 | 3.07E-08 |
| hsa-miR-106b-5p | MIRT138358 | FRMD6 | 2.69 | 3.07E-08 |
| hsa-miR-181a-5p | MIRT138427 | KIF2C | 2.82 | 1.11E-11 |
| hsa-miR-181b-5p | MIRT138428 | KIF2C | 2.82 | 1.11E-11 |
| hsa-miR-17-5p | MIRT147272 | KPNA2 | 2.15 | 1.09E-08 |
| hsa-miR-106b-5p | MIRT147283 | KPNA2 | 2.15 | 1.09E-08 |
| hsa-miR-27a-3p | MIRT148810 | PMAIP1 | 2.60 | 1.81E-09 |
| hsa-miR-182-3p | MIRT162043 | TFRC | 2.08 | 1.18E-04 |
| hsa-miR-17-3p | MIRT180553 | TXNIP | -2.06 | 1.05E-05 |
| hsa-miR-25-3p | MIRT243158 | SOX11 | 3.46 | 1.80E-05 |
| hsa-miR-25-3p | MIRT250939 | CDK5R1 | 2.32 | 1.52E-04 |
| hsa-miR-181a-5p | MIRT252248 | PMAIP1 | 2.60 | 1.81E-09 |
| hsa-miR-181b-5p | MIRT252249 | PMAIP1 | 2.60 | 1.81E-09 |
| hsa-miR-17-5p | MIRT266047 | FJX1 | 2.77 | 1.25E-08 |
| hsa-miR-93-5p | MIRT266050 | FJX1 | 2.77 | 1.25E-08 |
| hsa-miR-106b-5p | MIRT266053 | FJX1 | 2.77 | 1.25E-08 |
| hsa-miR-18a-5p | MIRT273157 | RAD51AP1 | 2.58 | 7.27E-08 |
| hsa-miR-27a-3p | MIRT320050 | EN2 | 4.26 | 6.82E-05 |
| hsa-miR-106b-5p | MIRT437468 | RUNX3 | 2.36 | 5.32E-06 |
| hsa-miR-223-3p | MIRT437691 | ZNF365 | 2.33 | 7.27E-03 |
| hsa-miR-27a-3p | MIRT437697 | CAB39L | -2.24 | 2.10E-09 |
| hsa-miR-17-5p | MIRT437865 | TGFB1 | 2.19 | 5.73E-07 |
| hsa-miR-93-5p | MIRT438039 | TGFB1 | 2.19 | 5.73E-07 |
| hsa-miR-93-5p | MIRT438087 | CXCL8 | 4.02 | 3.14E-05 |
| hsa-miR-21-5p | MIRT438112 | CXCL10 | 3.39 | 1.79E-03 |
| hsa-miR-223-3p | MIRT438283 | CCL3 | 2.18 | 3.65E-04 |
| hsa-miR-181a-5p | MIRT438509 | TWIST1 | 2.84 | 2.08E-05 |
| hsa-miR-182-5p | MIRT438756 | ULBP2 | 4.80 | 9.71E-09 |
| hsa-miR-223-3p | MIRT438885 | CFTR | -3.04 | 9.36E-05 |
| hsa-miR-106b-5p | MIRT439935 | POLQ | 2.78 | 5.09E-09 |
| hsa-miR-106b-5p | MIRT439958 | PKMYT1 | 3.39 | 1.66E-11 |
| hsa-miR-21-5p | MIRT440420 | ITGB8 | 2.03 | 8.45E-06 |
| hsa-miR-106b-5p | MIRT440757 | E2F2 | 2.04 | 6.30E-06 |
| hsa-miR-18a-5p | MIRT442747 | PTCHD1 | -2.87 | 2.08E-05 |
| hsa-miR-182-3p | MIRT442881 | ZC4H2 | -2.39 | 3.60E-07 |
| hsa-miR-93-5p | MIRT443254 | A1CF | -4.09 | 1.32E-10 |
| hsa-miR-17-5p | MIRT443259 | A1CF | -4.09 | 1.32E-10 |
| hsa-miR-106b-5p | MIRT443260 | A1CF | -4.09 | 1.32E-10 |
| hsa-miR-183-3p | MIRT444473 | WNT5A | 2.39 | 8.54E-04 |
| hsa-miR-183-3p | MIRT450043 | ARL4D | 2.85 | 5.16E-06 |
| hsa-miR-155-5p | MIRT450858 | HHIP | -2.48 | 1.60E-03 |
| hsa-miR-17-5p | MIRT450914 | CADM2 | -4.38 | 2.37E-08 |
| hsa-miR-93-5p | MIRT450918 | CADM2 | -4.38 | 2.37E-08 |
| hsa-miR-106b-5p | MIRT450921 | CADM2 | -4.38 | 2.37E-08 |
| hsa-miR-182-5p | MIRT452673 | GPR156 | 2.30 | 2.31E-03 |
| hsa-miR-484 | MIRT452995 | CABP4 | -2.00 | 6.28E-06 |
| hsa-miR-25-5p | MIRT453804 | KBTBD12 | -4.72 | 7.62E-13 |
| hsa-miR-25-3p | MIRT456013 | CYP2C19 | -2.57 | 1.49E-04 |
| hsa-miR-25-5p | MIRT458506 | HASPIN | 2.08 | 5.77E-07 |
| hsa-miR-25-5p | MIRT462534 | PLLP | -2.41 | 8.32E-09 |
| hsa-miR-23a-3p | MIRT462564 | STS | -2.05 | 7.73E-12 |
| hsa-miR-23a-5p | MIRT463884 | WNT7B | 5.99 | 2.65E-09 |
| hsa-miR-18a-3p | MIRT464908 | TXNIP | -2.06 | 1.05E-05 |
| hsa-miR-155-3p | MIRT464916 | TXNIP | -2.06 | 1.05E-05 |
| hsa-miR-18a-5p | MIRT464927 | TXNIP | -2.06 | 1.05E-05 |
| hsa-miR-27a-3p | MIRT466403 | TGFBR3 | -2.15 | 1.91E-04 |
| hsa-miR-223-3p | MIRT467584 | SLC7A5 | 2.20 | 4.35E-05 |
| hsa-miR-182-5p | MIRT468529 | SERPINH1 | 2.05 | 2.08E-05 |
| hsa-miR-106b-5p | MIRT472340 | NETO2 | 2.11 | 1.42E-05 |
| hsa-miR-17-5p | MIRT472342 | NETO2 | 2.11 | 1.42E-05 |
| hsa-miR-93-5p | MIRT472343 | NETO2 | 2.11 | 1.42E-05 |
| hsa-miR-132-3p | MIRT476656 | FZD6 | 2.10 | 1.73E-08 |
| hsa-miR-25-5p | MIRT477149 | FADS1 | 2.02 | 2.83E-03 |
| hsa-miR-25-5p | MIRT479714 | CCNF | 2.03 | 1.02E-07 |
| hsa-miR-505-5p | MIRT482744 | HES7 | 3.14 | 2.35E-07 |
| hsa-miR-484 | MIRT487267 | CCNF | 2.03 | 1.02E-07 |
| hsa-miR-132-5p | MIRT490730 | SLC9A3 | -3.89 | 7.69E-08 |
| hsa-miR-93-5p | MIRT493614 | HMGB3 | 2.41 | 2.90E-08 |
| hsa-miR-17-5p | MIRT493617 | HMGB3 | 2.41 | 2.90E-08 |
| hsa-miR-17-3p | MIRT495941 | MYRF | -4.95 | 2.77E-18 |
| hsa-miR-505-5p | MIRT496610 | POU6F2 | 3.32 | 8.18E-03 |
| hsa-miR-23a-5p | MIRT500353 | ZNF385A | 2.69 | 1.05E-09 |
| hsa-miR-93-5p | MIRT502401 | GATA6 | -3.91 | 6.22E-14 |
| hsa-miR-17-5p | MIRT502405 | GATA6 | -3.91 | 6.22E-14 |
| hsa-miR-106b-5p | MIRT502406 | GATA6 | -3.91 | 6.22E-14 |
| hsa-miR-25-3p | MIRT503127 | BCL11B | 2.83 | 2.08E-05 |
| hsa-miR-505-3p | MIRT503206 | ACER2 | -2.76 | 2.96E-13 |
| hsa-miR-106b-5p | MIRT503212 | ACER2 | -2.76 | 2.96E-13 |
| hsa-miR-17-5p | MIRT503216 | ACER2 | -2.76 | 2.96E-13 |
| hsa-miR-93-5p | MIRT503219 | ACER2 | -2.76 | 2.96E-13 |
| hsa-miR-182-5p | MIRT503220 | ACER2 | -2.76 | 2.96E-13 |
| hsa-miR-27a-3p | MIRT503224 | ACER2 | -2.76 | 2.96E-13 |
| hsa-miR-505-5p | MIRT503236 | C16orf74 | 2.91 | 2.10E-06 |
| hsa-miR-21-5p | MIRT503919 | FBXL13 | -2.06 | 2.08E-05 |
| hsa-miR-505-3p | MIRT504487 | SOX11 | 3.46 | 1.80E-05 |
| hsa-miR-21-3p | MIRT504490 | SOX11 | 3.46 | 1.80E-05 |
| hsa-miR-223-5p | MIRT504721 | PLEKHA6 | -4.00 | 1.56E-14 |
| hsa-miR-183-3p | MIRT504766 | CYSTM1 | -3.66 | 4.44E-17 |
| hsa-miR-17-5p | MIRT506849 | KIF23 | 2.71 | 2.19E-11 |
| hsa-miR-93-5p | MIRT506852 | KIF23 | 2.71 | 2.19E-11 |
| hsa-miR-93-5p | MIRT506883 | PCLAF | 2.57 | 4.58E-09 |
| hsa-miR-17-5p | MIRT506888 | PCLAF | 2.57 | 4.58E-09 |
| hsa-miR-106b-5p | MIRT506889 | PCLAF | 2.57 | 4.58E-09 |
| hsa-miR-223-5p | MIRT506906 | KCNJ3 | -4.34 | 4.08E-09 |
| hsa-miR-223-5p | MIRT508363 | ASB11 | -5.42 | 5.26E-11 |
| hsa-miR-183-5p | MIRT508455 | SHOX2 | 3.54 | 1.18E-05 |
| hsa-miR-93-5p | MIRT508833 | GPR155 | -2.53 | 9.60E-07 |
| hsa-miR-17-5p | MIRT508838 | GPR155 | -2.53 | 9.60E-07 |
| hsa-miR-106b-5p | MIRT508839 | GPR155 | -2.53 | 9.60E-07 |
| hsa-miR-223-5p | MIRT509244 | OTX1 | 2.57 | 2.27E-05 |
| hsa-miR-25-3p | MIRT510442 | ZIC5 | 4.44 | 3.89E-04 |
| hsa-miR-25-3p | MIRT510543 | XKR7 | -2.30 | 1.13E-04 |
| hsa-miR-223-5p | MIRT510960 | PMAIP1 | 2.60 | 1.81E-09 |
| hsa-miR-23a-5p | MIRT511566 | HIST3H2BB | 2.41 | 2.52E-04 |
| hsa-miR-27a-3p | MIRT511593 | HIST2H2AA3 | 2.04 | 4.09E-04 |
| hsa-miR-181b-3p | MIRT512972 | PPP1R14C | 4.67 | 5.08E-08 |
| hsa-miR-505-3p | MIRT513076 | IL20RB | 3.66 | 3.26E-04 |
| hsa-miR-181b-3p | MIRT513264 | SCUBE1 | -2.87 | 9.03E-05 |
| hsa-miR-223-5p | MIRT514981 | CABP4 | -2.00 | 6.28E-06 |
| hsa-miR-223-5p | MIRT515407 | WDR72 | 3.69 | 3.92E-06 |
| hsa-miR-18a-3p | MIRT515910 | MFAP2 | 3.79 | 2.65E-08 |
| hsa-miR-223-5p | MIRT517271 | ANG | -2.48 | 3.29E-09 |
| hsa-miR-484 | MIRT518171 | FGB | -4.09 | 2.13E-04 |
| hsa-miR-223-5p | MIRT518655 | SLC2A12 | -2.06 | 1.04E-03 |
| hsa-miR-93-5p | MIRT518968 | GRK7 | 2.23 | 1.83E-04 |
| hsa-miR-17-5p | MIRT518973 | GRK7 | 2.23 | 1.83E-04 |
| hsa-miR-106b-5p | MIRT518974 | GRK7 | 2.23 | 1.83E-04 |
| hsa-miR-93-5p | MIRT519428 | KCNA7 | 2.70 | 2.20E-06 |
| hsa-miR-17-5p | MIRT519433 | KCNA7 | 2.70 | 2.20E-06 |
| hsa-miR-106b-5p | MIRT519434 | KCNA7 | 2.70 | 2.20E-06 |
| hsa-miR-93-5p | MIRT519544 | TMEM38A | -2.01 | 1.07E-06 |
| hsa-miR-17-5p | MIRT519549 | TMEM38A | -2.01 | 1.07E-06 |
| hsa-miR-106b-5p | MIRT519550 | TMEM38A | -2.01 | 1.07E-06 |
| hsa-miR-223-5p | MIRT522843 | NEXMIF | -3.02 | 2.12E-04 |
| hsa-miR-27a-5p | MIRT523292 | HIST1H1E | 2.29 | 4.72E-06 |
| hsa-miR-223-5p | MIRT523312 | HHIP | -2.48 | 1.60E-03 |
| hsa-miR-223-5p | MIRT523389 | GRIN2B | -2.54 | 2.92E-06 |
| hsa-miR-223-5p | MIRT523888 | EPHA5 | -3.16 | 1.02E-04 |
| hsa-miR-183-3p | MIRT525349 | XKR7 | -2.30 | 1.13E-04 |
| hsa-miR-505-3p | MIRT526131 | GJB7 | 3.38 | 2.08E-05 |
| hsa-miR-181b-5p | MIRT526144 | GJB7 | 3.38 | 2.08E-05 |
| hsa-miR-181a-5p | MIRT526145 | GJB7 | 3.38 | 2.08E-05 |
| hsa-miR-93-5p | MIRT527255 | TMEM196 | -2.21 | 8.30E-04 |
| hsa-miR-17-5p | MIRT527260 | TMEM196 | -2.21 | 8.30E-04 |
| hsa-miR-106b-5p | MIRT527261 | TMEM196 | -2.21 | 8.30E-04 |
| hsa-miR-181b-3p | MIRT529502 | IYD | -4.78 | 2.42E-10 |
| hsa-miR-93-3p | MIRT530008 | FAM174B | -2.42 | 2.93E-10 |
| hsa-miR-18a-3p | MIRT530776 | GPD1 | -3.17 | 2.78E-12 |
| hsa-miR-23a-3p | MIRT531701 | ADAM28 | -3.14 | 2.49E-09 |
| hsa-miR-505-3p | MIRT532095 | SYT4 | -4.31 | 6.66E-08 |
| hsa-miR-132-3p | MIRT532420 | ART4 | -2.12 | 2.87E-05 |
| hsa-miR-106b-5p | MIRT532926 | ZNF385A | 2.69 | 1.05E-09 |
| hsa-miR-17-5p | MIRT532928 | ZNF385A | 2.69 | 1.05E-09 |
| hsa-miR-93-5p | MIRT532929 | ZNF385A | 2.69 | 1.05E-09 |
| hsa-miR-27a-5p | MIRT533141 | WNT10A | 2.79 | 1.47E-03 |
| hsa-miR-17-3p | MIRT534808 | RAB37 | -2.16 | 1.83E-08 |
| hsa-miR-181b-5p | MIRT536907 | HEPHL1 | 4.83 | 2.05E-04 |
| hsa-miR-181a-5p | MIRT536908 | HEPHL1 | 4.83 | 2.05E-04 |
| hsa-miR-23a-5p | MIRT540225 | SAMD5 | -2.45 | 4.02E-04 |
| hsa-miR-25-3p | MIRT541471 | AURKA | 2.09 | 4.29E-09 |
| hsa-miR-25-3p | MIRT542928 | HOXC8 | 4.59 | 2.27E-10 |
| hsa-miR-223-5p | MIRT545025 | PLP1 | -3.36 | 7.96E-05 |
| hsa-miR-25-3p | MIRT545263 | TRIM36 | -2.44 | 1.48E-07 |
| hsa-miR-132-3p | MIRT546160 | TSPAN12 | -3.05 | 8.27E-13 |
| hsa-miR-132-3p | MIRT547614 | LIFR | -3.04 | 1.01E-07 |
| hsa-miR-183-3p | MIRT547734 | KIF23 | 2.71 | 2.19E-11 |
| hsa-miR-25-3p | MIRT548125 | GATA6 | -3.91 | 6.22E-14 |
| hsa-miR-25-3p | MIRT550539 | MYZAP | -3.62 | 2.36E-09 |
| hsa-miR-223-5p | MIRT550944 | CTSE | -9.34 | 1.57E-17 |
| hsa-miR-25-3p | MIRT551224 | CIDEC | -4.59 | 7.62E-13 |
| hsa-miR-223-5p | MIRT551414 | LRRC31 | -5.48 | 1.04E-10 |
| hsa-miR-223-5p | MIRT551505 | ADH1B | -5.84 | 7.70E-10 |
| hsa-miR-106b-5p | MIRT553574 | TMEM100 | -3.70 | 5.79E-11 |
| hsa-miR-17-5p | MIRT553575 | TMEM100 | -3.70 | 5.79E-11 |
| hsa-miR-93-5p | MIRT554412 | SCD | 2.02 | 1.70E-04 |
| hsa-miR-17-5p | MIRT554417 | SCD | 2.02 | 1.70E-04 |
| hsa-miR-106b-5p | MIRT554418 | SCD | 2.02 | 1.70E-04 |
| hsa-miR-25-3p | MIRT555828 | PAX9 | 3.42 | 5.57E-04 |
| hsa-miR-25-3p | MIRT556430 | LONRF3 | -2.82 | 2.07E-07 |
| hsa-miR-223-5p | MIRT556565 | LIFR | -3.04 | 1.01E-07 |
| hsa-miR-27a-3p | MIRT557017 | HOXD11 | 6.75 | 1.17E-07 |
| hsa-miR-93-5p | MIRT557030 | HOXD11 | 6.75 | 1.17E-07 |
| hsa-miR-106b-5p | MIRT557032 | HOXD11 | 6.75 | 1.17E-07 |
| hsa-miR-17-5p | MIRT557033 | HOXD11 | 6.75 | 1.17E-07 |
| hsa-miR-181b-5p | MIRT557275 | HMGA2 | 3.34 | 2.63E-03 |
| hsa-miR-181a-5p | MIRT557276 | HMGA2 | 3.34 | 2.63E-03 |
| hsa-miR-182-5p | MIRT558452 | DDAH1 | -2.69 | 3.65E-10 |
| hsa-miR-183-5p | MIRT558453 | DDAH1 | -2.69 | 3.65E-10 |
| hsa-miR-183-5p | MIRT559287 | AURKA | 2.09 | 4.29E-09 |
| hsa-miR-17-5p | MIRT561651 | RUNX3 | 2.36 | 5.32E-06 |
| hsa-miR-23a-3p | MIRT562011 | LMNB1 | 2.05 | 6.26E-08 |
| hsa-miR-93-5p | MIRT563379 | DSPP | 4.53 | 1.27E-10 |
| hsa-miR-17-5p | MIRT563384 | DSPP | 4.53 | 1.27E-10 |
| hsa-miR-106b-5p | MIRT563385 | DSPP | 4.53 | 1.27E-10 |
| hsa-miR-93-5p | MIRT567057 | KCNB1 | -3.39 | 2.08E-05 |
| hsa-miR-106b-5p | MIRT567058 | KCNB1 | -3.39 | 2.08E-05 |
| hsa-miR-17-5p | MIRT567062 | KCNB1 | -3.39 | 2.08E-05 |
| hsa-miR-25-5p | MIRT568866 | LY6H | -3.02 | 1.22E-06 |
| hsa-miR-223-5p | MIRT570522 | SHH | -2.90 | 1.05E-03 |
| hsa-miR-93-5p | MIRT571015 | CKAP2 | 2.35 | 2.19E-10 |
| hsa-miR-17-5p | MIRT571020 | CKAP2 | 2.35 | 2.19E-10 |
| hsa-miR-106b-5p | MIRT571021 | CKAP2 | 2.35 | 2.19E-10 |
| hsa-miR-183-3p | MIRT573287 | DLX4 | 3.39 | 7.47E-08 |
| hsa-miR-223-5p | MIRT574251 | G6PC | -2.32 | 1.71E-04 |
| hsa-miR-223-5p | MIRT606947 | ARRB1 | -2.77 | 1.30E-14 |
| hsa-miR-17-3p | MIRT613399 | DNAH17 | 2.68 | 1.53E-03 |
| hsa-miR-183-3p | MIRT615698 | NEGR1 | -2.45 | 2.89E-05 |
| hsa-miR-183-3p | MIRT615983 | FSTL4 | 3.34 | 1.43E-04 |
| hsa-miR-93-5p | MIRT620977 | TM4SF5 | -6.37 | 2.44E-19 |
| hsa-miR-17-5p | MIRT620982 | TM4SF5 | -6.37 | 2.44E-19 |
| hsa-miR-106b-5p | MIRT620983 | TM4SF5 | -6.37 | 2.44E-19 |
| hsa-miR-183-3p | MIRT622601 | PROM1 | -4.55 | 1.51E-08 |
| hsa-miR-23a-3p | MIRT624024 | EN2 | 4.26 | 6.82E-05 |
| hsa-miR-505-5p | MIRT624874 | ABCC12 | -3.48 | 3.62E-08 |
| hsa-miR-23a-5p | MIRT626016 | XRCC2 | 2.52 | 1.62E-08 |
| hsa-miR-23a-5p | MIRT626814 | PRR11 | 2.70 | 3.59E-09 |
| hsa-miR-93-5p | MIRT626930 | HIST1H2BG | 3.00 | 2.29E-05 |
| hsa-miR-17-5p | MIRT626935 | HIST1H2BG | 3.00 | 2.29E-05 |
| hsa-miR-106b-5p | MIRT626936 | HIST1H2BG | 3.00 | 2.29E-05 |
| hsa-miR-223-5p | MIRT628115 | IFFO2 | 2.09 | 8.63E-05 |
| hsa-miR-93-5p | MIRT628565 | MELK | 2.91 | 4.25E-10 |
| hsa-miR-17-5p | MIRT628570 | MELK | 2.91 | 4.25E-10 |
| hsa-miR-106b-5p | MIRT628571 | MELK | 2.91 | 4.25E-10 |
| hsa-miR-25-3p | MIRT632579 | POLQ | 2.78 | 5.09E-09 |
| hsa-miR-181b-5p | MIRT633492 | WDR72 | 3.69 | 3.92E-06 |
| hsa-miR-181a-5p | MIRT633493 | WDR72 | 3.69 | 3.92E-06 |
| hsa-miR-93-5p | MIRT634098 | APOH | -3.61 | 1.82E-06 |
| hsa-miR-17-5p | MIRT634103 | APOH | -3.61 | 1.82E-06 |
| hsa-miR-106b-5p | MIRT634104 | APOH | -3.61 | 1.82E-06 |
| hsa-miR-18a-3p | MIRT634403 | PLEKHA6 | -4.00 | 1.56E-14 |
| hsa-miR-27a-5p | MIRT639522 | ACER2 | -2.76 | 2.96E-13 |
| hsa-miR-27a-5p | MIRT640810 | ZMAT1 | -2.69 | 2.20E-06 |
| hsa-miR-27a-3p | MIRT641998 | NR2F2 | -2.11 | 1.77E-09 |
| hsa-miR-181b-3p | MIRT642414 | CILP2 | 2.86 | 1.22E-04 |
| hsa-miR-23a-3p | MIRT642995 | SNTG1 | -2.00 | 2.59E-03 |
| hsa-miR-484 | MIRT643427 | ERVMER34-1 | 3.08 | 7.37E-06 |
| hsa-miR-23a-5p | MIRT645121 | HES2 | 4.92 | 5.43E-06 |
| hsa-miR-18a-3p | MIRT645792 | PLCE1 | -2.05 | 2.17E-09 |
| hsa-miR-484 | MIRT645866 | GBP6 | 5.53 | 7.23E-05 |
| hsa-miR-23a-3p | MIRT646026 | S100A7A | 6.65 | 1.79E-03 |
| hsa-miR-223-5p | MIRT647869 | HEPHL1 | 4.83 | 2.05E-04 |
| hsa-miR-505-3p | MIRT650341 | TREM1 | 3.55 | 6.55E-05 |
| hsa-miR-484 | MIRT650927 | ST6GALNAC1 | -4.17 | 3.08E-10 |
| hsa-miR-155-3p | MIRT651489 | WT1 | 2.86 | 3.48E-03 |
| hsa-miR-181a-3p | MIRT654721 | PRR11 | 2.70 | 3.59E-09 |
| hsa-miR-23a-5p | MIRT660619 | ANKS4B | -4.14 | 3.28E-10 |
| hsa-miR-484 | MIRT663265 | ATP1A2 | -3.07 | 8.91E-04 |
| hsa-miR-25-5p | MIRT664256 | NMUR1 | -2.50 | 8.73E-06 |
| hsa-miR-183-5p | MIRT664965 | TDRD1 | -3.09 | 5.66E-05 |
| hsa-miR-181b-5p | MIRT669767 | ZNF556 | 2.24 | 9.50E-04 |
| hsa-miR-181a-5p | MIRT669768 | ZNF556 | 2.24 | 9.50E-04 |
| hsa-miR-181b-3p | MIRT669772 | CNDP1 | -2.09 | 2.08E-05 |
| hsa-miR-17-3p | MIRT670719 | SEMA3E | -2.66 | 2.39E-03 |
| hsa-miR-183-5p | MIRT672296 | GP2 | -5.62 | 1.97E-07 |
| hsa-miR-18a-3p | MIRT673109 | MFSD2A | 2.27 | 1.05E-04 |
| hsa-miR-93-5p | MIRT673134 | MFSD2A | 2.27 | 1.05E-04 |
| hsa-miR-17-5p | MIRT673139 | MFSD2A | 2.27 | 1.05E-04 |
| hsa-miR-106b-5p | MIRT673140 | MFSD2A | 2.27 | 1.05E-04 |
| hsa-miR-23a-5p | MIRT675391 | SVOP | -2.67 | 1.76E-07 |
| hsa-miR-484 | MIRT676262 | PBOV1 | -2.31 | 1.00E-04 |
| hsa-miR-23a-5p | MIRT678144 | SLC4A4 | -5.64 | 8.47E-21 |
| hsa-miR-23a-5p | MIRT679741 | CABP4 | -2.00 | 6.28E-06 |
| hsa-miR-23a-5p | MIRT679976 | E2F2 | 2.04 | 6.30E-06 |
| hsa-miR-27a-3p | MIRT682416 | OLR1 | 3.26 | 1.71E-04 |
| hsa-miR-182-3p | MIRT682588 | CPA4 | 3.44 | 5.27E-03 |
| hsa-miR-93-5p | MIRT683989 | QRFPR | 4.54 | 4.14E-03 |
| hsa-miR-17-5p | MIRT683994 | QRFPR | 4.54 | 4.14E-03 |
| hsa-miR-106b-5p | MIRT683995 | QRFPR | 4.54 | 4.14E-03 |
| hsa-miR-93-5p | MIRT684660 | PDE4C | -3.46 | 7.46E-12 |
| hsa-miR-17-5p | MIRT684665 | PDE4C | -3.46 | 7.46E-12 |
| hsa-miR-106b-5p | MIRT684666 | PDE4C | -3.46 | 7.46E-12 |
| hsa-miR-93-5p | MIRT685967 | PTGIS | -2.71 | 9.65E-04 |
| hsa-miR-17-5p | MIRT685972 | PTGIS | -2.71 | 9.65E-04 |
| hsa-miR-106b-5p | MIRT685973 | PTGIS | -2.71 | 9.65E-04 |
| hsa-miR-93-5p | MIRT686118 | TNIP3 | 2.32 | 1.75E-03 |
| hsa-miR-17-5p | MIRT686123 | TNIP3 | 2.32 | 1.75E-03 |
| hsa-miR-106b-5p | MIRT686124 | TNIP3 | 2.32 | 1.75E-03 |
| hsa-miR-23a-5p | MIRT688733 | CNDP1 | -2.09 | 2.08E-05 |
| hsa-miR-23a-5p | MIRT689031 | ANGPTL3 | -2.97 | 8.32E-08 |
| hsa-miR-93-5p | MIRT689856 | HIST1H2BJ | 2.62 | 5.61E-05 |
| hsa-miR-17-5p | MIRT689861 | HIST1H2BJ | 2.62 | 5.61E-05 |
| hsa-miR-106b-5p | MIRT689862 | HIST1H2BJ | 2.62 | 5.61E-05 |
| hsa-miR-182-5p | MIRT689956 | ZNF185 | 2.13 | 2.46E-03 |
| hsa-miR-93-5p | MIRT694951 | ANKS4B | -4.14 | 3.28E-10 |
| hsa-miR-17-5p | MIRT694956 | ANKS4B | -4.14 | 3.28E-10 |
| hsa-miR-106b-5p | MIRT694957 | ANKS4B | -4.14 | 3.28E-10 |
| hsa-miR-155-3p | MIRT696720 | WNT3 | 2.46 | 2.34E-06 |
| hsa-miR-93-5p | MIRT697261 | ZYG11A | 2.56 | 7.88E-04 |
| hsa-miR-17-5p | MIRT697266 | ZYG11A | 2.56 | 7.88E-04 |
| hsa-miR-106b-5p | MIRT697267 | ZYG11A | 2.56 | 7.88E-04 |
| hsa-miR-17-3p | MIRT698540 | TFRC | 2.08 | 1.18E-04 |
| hsa-miR-505-3p | MIRT701491 | NEGR1 | -2.45 | 2.89E-05 |
| hsa-miR-93-5p | MIRT704213 | LDHD | -2.69 | 1.21E-06 |
| hsa-miR-17-5p | MIRT704218 | LDHD | -2.69 | 1.21E-06 |
| hsa-miR-106b-5p | MIRT704219 | LDHD | -2.69 | 1.21E-06 |
| hsa-miR-505-3p | MIRT704711 | CHEK1 | 2.01 | 1.08E-08 |
| hsa-miR-93-5p | MIRT705365 | ATP1B3 | 2.27 | 2.23E-07 |
| hsa-miR-17-5p | MIRT705370 | ATP1B3 | 2.27 | 2.23E-07 |
| hsa-miR-106b-5p | MIRT705371 | ATP1B3 | 2.27 | 2.23E-07 |
| hsa-miR-484 | MIRT706714 | GPR155 | -2.53 | 9.60E-07 |
| hsa-miR-181a-3p | MIRT708792 | CFHR3 | 2.33 | 9.38E-03 |
| hsa-miR-18a-3p | MIRT708995 | CABP4 | -2.00 | 6.28E-06 |
| hsa-miR-23a-3p | MIRT709247 | CENPM | 2.36 | 4.31E-08 |
| hsa-miR-155-5p | MIRT709796 | FOXE1 | 6.72 | 7.69E-06 |
| hsa-miR-505-5p | MIRT709797 | FOXE1 | 6.72 | 7.69E-06 |
| hsa-miR-183-3p | MIRT709867 | A1CF | -4.09 | 1.32E-10 |
| hsa-miR-93-3p | MIRT709918 | GRIK3 | -2.95 | 1.08E-03 |
| hsa-miR-132-5p | MIRT710087 | S100A9 | 4.66 | 1.13E-03 |
| hsa-miR-18a-3p | MIRT710270 | FAM107A | -3.98 | 4.19E-11 |
| hsa-miR-223-5p | MIRT710382 | PARD6G | 2.61 | 5.98E-07 |
| hsa-miR-106b-3p | MIRT711698 | GMPR | -2.57 | 3.77E-07 |
| hsa-miR-93-3p | MIRT716717 | SCN7A | -3.33 | 2.92E-05 |
| hsa-miR-93-3p | MIRT717236 | SH2D5 | 4.23 | 3.07E-05 |
| hsa-miR-23a-5p | MIRT718829 | CDT1 | 2.77 | 6.41E-10 |
| hsa-miR-505-3p | MIRT719661 | DMRT2 | 3.84 | 5.49E-03 |
| hsa-miR-183-5p | MIRT721372 | SEC14L6 | -2.58 | 7.43E-06 |
| hsa-miR-181b-3p | MIRT721578 | SLC5A12 | 4.39 | 7.72E-07 |
| hsa-miR-505-3p | MIRT721580 | SLC5A12 | 4.39 | 7.72E-07 |
| hsa-miR-93-3p | MIRT725341 | NEUROD1 | -3.92 | 9.62E-07 |
| hsa-miR-181b-5p | MIRT725541 | EN2 | 4.26 | 6.82E-05 |
| hsa-miR-181a-5p | MIRT725542 | EN2 | 4.26 | 6.82E-05 |
| hsa-miR-181b-5p | MIRT726332 | TFRC | 2.08 | 1.18E-04 |
| hsa-miR-181a-5p | MIRT726333 | TFRC | 2.08 | 1.18E-04 |
| hsa-miR-93-5p | MIRT726903 | POLQ | 2.78 | 5.09E-09 |
| hsa-miR-17-5p | MIRT726906 | POLQ | 2.78 | 5.09E-09 |
| hsa-miR-93-5p | MIRT726927 | PKMYT1 | 3.39 | 1.66E-11 |
| hsa-miR-17-5p | MIRT726928 | PKMYT1 | 3.39 | 1.66E-11 |
| hsa-miR-181b-5p | MIRT727098 | NCAPG | 2.72 | 9.02E-10 |
| hsa-miR-181a-5p | MIRT727100 | NCAPG | 2.72 | 9.02E-10 |
| hsa-miR-181b-5p | MIRT727458 | IL1A | 5.31 | 1.11E-04 |
| hsa-miR-181a-5p | MIRT727459 | IL1A | 5.31 | 1.11E-04 |
| hsa-miR-27a-3p | MIRT727818 | E2F7 | 3.47 | 4.11E-10 |
| hsa-miR-93-5p | MIRT727821 | E2F2 | 2.04 | 6.30E-06 |
| hsa-miR-17-5p | MIRT727822 | E2F2 | 2.04 | 6.30E-06 |
| hsa-miR-181a-5p | MIRT731403 | TUSC3 | 2.28 | 3.47E-04 |
| hsa-miR-27a-3p | MIRT731607 | 2 | 2.12 | 2.14E-03 |
| hsa-miR-106b-5p | MIRT732202 | PRRX1 | 2.47 | 3.16E-04 |
| hsa-miR-181b-5p | MIRT732307 | SPP1 | 4.88 | 8.05E-05 |
| hsa-miR-23a-3p | MIRT732483 | HNF1B | -6.70 | 8.71E-22 |
| hsa-miR-23a-3p | MIRT732488 | FOXM1 | 2.93 | 5.30E-09 |
| hsa-miR-23a-3p | MIRT732489 | HRG | 2.61 | 1.31E-03 |
| hsa-miR-17-5p | MIRT732631 | NTN4 | -2.15 | 5.86E-07 |
| hsa-miR-106b-5p | MIRT732724 | SLC2A4 | -2.66 | 1.19E-04 |
| hsa-miR-182-5p | MIRT732738 | PDK4 | -5.07 | 9.99E-14 |
| hsa-miR-25-3p | MIRT732909 | TNFSF10 | 2.31 | 3.09E-05 |
| hsa-miR-181a-5p | MIRT733003 | ALDH1A1 | -3.07 | 3.53E-04 |
| hsa-miR-21-5p | MIRT733174 | FZD6 | 2.10 | 1.73E-08 |
| hsa-miR-93-5p | MIRT733293 | ANG | -2.48 | 3.29E-09 |
| hsa-miR-23a-3p | MIRT733333 | GJA1 | 2.44 | 2.87E-04 |
| hsa-miR-223-3p | MIRT733398 | MAFB | 2.08 | 2.08E-05 |
| hsa-miR-182-3p | MIRT733403 | NOX4 | 2.92 | 1.33E-06 |
| hsa-miR-21-3p | MIRT733453 | HPGD | -4.09 | 9.00E-11 |
| hsa-miR-183-5p | MIRT733502 | PCLAF | 2.57 | 4.58E-09 |
| hsa-miR-25-3p | MIRT733741 | COL1A2 | 2.33 | 3.01E-03 |
| hsa-miR-132-3p | MIRT733764 | MMP9 | 3.33 | 2.12E-04 |
| hsa-miR-23a-3p | MIRT733809 | TRIM63 | -2.56 | 4.09E-04 |
| hsa-miR-223-5p | MIRT733966 | EGF | 3.35 | 3.10E-04 |
| hsa-miR-23a-5p | MIRT734092 | GJA1 | 2.44 | 2.87E-04 |
| hsa-miR-21-5p | MIRT734132 | FOXP3 | 2.12 | 2.08E-05 |
| hsa-miR-21-5p | MIRT734186 | MSLN | -3.05 | 3.66E-04 |
| hsa-miR-18a-5p | MIRT734267 | NR1I2 | -3.49 | 5.30E-07 |
| hsa-miR-181a-5p | MIRT734362 | PRKN | -2.45 | 1.15E-08 |
| hsa-miR-93-3p | MIRT734847 | MMP3 | 4.05 | 4.77E-04 |
| hsa-miR-93-5p | MIRT735341 | MMP3 | 4.05 | 4.77E-04 |
| hsa-miR-132-3p | MIRT735435 | SLC2A1 | 3.58 | 7.69E-07 |
| hsa-miR-132-5p | MIRT737409 | SLC7A5 | 2.20 | 4.35E-05 |
| hsa-miR-17-3p | MIRT737997 | HMGA2 | 3.34 | 2.63E-03 |
| hsa-miR-183-5p | MIRT738182 | PLK1 | 2.48 | 2.14E-08 |
| hsa-miR-18a-3p | MIRT738296 | SH2D5 | 4.23 | 3.07E-05 |
| hsa-miR-23a-5p | MIRT739189 | IFFO2 | 2.09 | 8.63E-05 |
| hsa-miR-25-3p | MIRT739350 | CCNB1 | 2.69 | 6.89E-11 |
| hsa-miR-25-3p | MIRT739353 | CHST1 | 3.01 | 3.77E-04 |
| hsa-miR-25-3p | MIRT739359 | ITGB8 | 2.03 | 8.45E-06 |
| hsa-miR-25-3p | MIRT739366 | RAD51 | 2.49 | 3.57E-09 |
| hsa-miR-25-5p | MIRT739381 | KIAA1456 | -2.56 | 9.86E-05 |
| hsa-miR-27a-3p | MIRT739444 | HOXC11 | 3.99 | 1.01E-05 |
| hsa-miR-484 | MIRT749051 | SLC7A5 | 2.20 | 4.35E-05 |
| hsa-miR-505-3p | MIRT749574 | SCD | 2.02 | 1.70E-04 |
| hsa-miR-505-5p | MIRT749587 | PLK1 | 2.48 | 2.14E-08 |
| hsa-miR-106b-5p | MIRT761133 | PMAIP1 | 2.60 | 1.81E-09 |
| hsa-miR-155-3p | MIRT762909 | CDHR1 | 3.74 | 9.70E-04 |
| hsa-miR-17-3p | MIRT762946 | ATOH8 | -3.35 | 1.53E-10 |
| hsa-miR-17-5p | MIRT762955 | PMAIP1 | 2.60 | 1.81E-09 |
| hsa-miR-181b-3p | MIRT762975 | APOBEC4 | -2.60 | 7.74E-06 |
| hsa-miR-182-5p | MIRT763002 | IL31RA | 2.95 | 3.94E-03 |
| hsa-miR-182-5p | MIRT763003 | LOX | 2.04 | 7.75E-04 |
| hsa-miR-23a-3p | MIRT764012 | TXNIP | -2.06 | 1.05E-05 |
| hsa-miR-23a-5p | MIRT764016 | CLDN16 | 3.41 | 5.78E-06 |
| hsa-miR-25-3p | MIRT764170 | GCNT3 | -2.64 | 3.26E-04 |
| hsa-miR-25-3p | MIRT764177 | PGPEP1 | -2.03 | 1.41E-09 |
| hsa-miR-25-5p | MIRT764204 | ASPA | -3.67 | 2.57E-10 |
| hsa-miR-25-5p | MIRT764215 | F2 | -2.62 | 1.70E-05 |
| hsa-miR-25-5p | MIRT764221 | GCNT3 | -2.64 | 3.26E-04 |
| hsa-miR-25-5p | MIRT764231 | KNL1 | 2.32 | 4.04E-07 |
| hsa-miR-25-5p | MIRT764251 | RPH3AL | -2.30 | 1.89E-06 |
| hsa-miR-505-3p | MIRT773096 | RIMS4 | -4.20 | 6.92E-07 |
| hsa-miR-93-5p | MIRT782763 | PMAIP1 | 2.60 | 1.81E-09 |
| hsa-miR-223-5p | MIRT784266 | INHBA | 3.87 | 2.53E-05 |
| hsa-miR-223-5p | MIRT784273 | RSPO1 | -2.22 | 1.08E-05 |
| hsa-miR-505-5p | MIRT790685 | GPER1 | -3.70 | 3.71E-15 |

**Supplemental Table 3:** Functional analysis of miRNA target genes identified 31 significantly enriched signaling pathways and Hallmark gene sets (BH-adjusted p-value < 0.05)

| **Gene Set** | **Gene Ratio** | **p-value** | **BH-adjusted p-value** |
| --- | --- | --- | --- |
| HALLMARK G2M CHECKPOINT | 33/379 | 5.65E-23 | 2.34E-20 |
| HALLMARK E2F TARGETS | 31/379 | 8.18E-21 | 2.77E-18 |
| KEGG PATHWAYS IN CANCER | 21/379 | 2.26E-07 | 9.82E-06 |
| HALLMARK EPITHELIAL MESENCHYMAL TRANSITION | 19/379 | 1.83E-09 | 1.21E-07 |
| HALLMARK GLYCOLYSIS | 14/379 | 9.65E-06 | 2.41E-04 |
| KEGG CELL CYCLE | 12/379 | 1.51E-06 | 4.99E-05 |
| HALLMARK INFLAMMATORY RESPONSE | 12/379 | 1.81E-04 | 2.66E-03 |
| HALLMARK MTORC1 SIGNALING | 12/379 | 1.81E-04 | 2.66E-03 |
| KEGG CYTOKINE CYTOKINE RECEPTOR INTERACTION | 12/379 | 2.15E-03 | 1.73E-02 |
| HALLMARK MITOTIC SPINDLE | 11/379 | 6.65E-04 | 7.41E-03 |
| HALLMARK ESTROGEN RESPONSE EARLY | 11/379 | 6.94E-04 | 7.64E-03 |
| KEGG BASAL CELL CARCINOMA | 10/379 | 2.81E-08 | 1.50E-06 |
| KEGG HEDGEHOG SIGNALING PATHWAY | 10/379 | 3.37E-08 | 1.77E-06 |
| HALLMARK ESTROGEN RESPONSE LATE | 10/379 | 2.43E-03 | 1.88E-02 |
| HALLMARK KRAS SIGNALING UP | 10/379 | 2.43E-03 | 1.88E-02 |
| HALLMARK P53 PATHWAY | 10/379 | 2.43E-03 | 1.88E-02 |
| HALLMARK UV RESPONSE DN | 9/379 | 8.56E-04 | 8.88E-03 |
| KEGG MELANOGENESIS | 8/379 | 3.45E-04 | 4.50E-03 |
| KEGG OOCYTE MEIOSIS | 8/379 | 6.91E-04 | 7.64E-03 |
| HALLMARK COAGULATION | 8/379 | 2.63E-03 | 1.99E-02 |
| KEGG WNT SIGNALING PATHWAY | 8/379 | 4.37E-03 | 2.92E-02 |
| KEGG CELL ADHESION MOLECULES CAMS | 7/379 | 8.03E-03 | 4.58E-02 |
| KEGG P53 SIGNALING PATHWAY | 6/379 | 1.08E-03 | 1.06E-02 |
| KEGG ECM RECEPTOR INTERACTION | 6/379 | 3.19E-03 | 2.29E-02 |
| KEGG PROGESTERONE MEDIATED OOCYTE MATURATION | 6/379 | 3.39E-03 | 2.39E-02 |
| HALLMARK ANGIOGENESIS | 5/379 | 3.48E-04 | 4.50E-03 |
| KEGG MELANOMA | 5/379 | 7.39E-03 | 4.34E-02 |
| KEGG PROXIMAL TUBULE BICARBONATE RECLAMATION | 4/379 | 5.77E-04 | 6.63E-03 |
| HALLMARK NOTCH SIGNALING | 4/379 | 2.08E-03 | 1.68E-02 |
| KEGG BLADDER CANCER | 4/379 | 5.65E-03 | 3.53E-02 |
| KEGG BIOSYNTHESIS OF UNSATURATED FATTY ACIDS | 3/379 | 6.01E-03 | 3.71E-02 |

**Supplemental Table 4:** miRNA panel selection and logistic regression model in serum biomarker prioritization and training phases.

| **miRNA** | **Serum biomarker prioritization phase** | | |  | **Training phase** | | | |
| --- | --- | --- | --- | --- | --- | --- | --- | --- |
|  | **Mean**  **CT value** | **ESCC vs. Healthy** | **AUC** |  | **Logistic regression model** | | | |
|  |  |  |  |  | **Estimate** | **Std.Error** | **z value** | **Pz(>IzI)** |
| Let-7d | 33.4 | n.s. |  |  |  |  |  |  |
| Let-7i | 32.1 | n.s. |  |  |  |  |  |  |
| **miR-103** | **32.5** | **Up** | **0.59** |  | **-1.375** | **0.79** | **-1.73** | **0.08** |
| **miR-106b** | **31.6** | **Up** | **0.60** |  | **0.454** | **0.55** | **0.82** | **0.40** |
| miR-132 | 34.8 | n.s. |  |  |  |  |  |  |
| **miR-151** | **32.5** | **Up** | **0.63** |  | **-3.278** | **1.43** | **-2.28** | **0.02** |
| miR-155 | 31.2 | n.s. |  |  |  |  |  |  |
| **miR-17** | **30.5** | **Up** | **0.78** |  | **3.753** | **0.81** | **4.63** | **0.000** |
| **miR-181a** | **33.9** | **Up** | **0.52** |  | **-8.505** | **3.69** | **-2.30** | **0.002** |
| miR-181b | 34.2 | n.s. |  |  |  |  |  |  |
| miR-182 | 38.8 | n.s. |  |  |  |  |  |  |
| miR-183 | 39.8 | n.s. |  |  |  |  |  |  |
| miR-18a | 36.9 | n.s. |  |  |  |  |  |  |
| **miR-21** | **29.4** | **Up** | **0.79** |  | **0.209** | **0.14** | **1.45** | **0.14** |
| miR-223 | 25.9 | Down |  |  |  |  |  |  |
| miR-23a | 34.4 | n.s. |  |  |  |  |  |  |
| **miR-25** | **31.9** | **Up** | **0.66** |  | **4.149** | **1.31** | **3.14** | **0.002** |
| miR-27a | 34.0 | n.s. |  |  |  |  |  |  |
| miR-484 | 28.1 | n.s. |  |  |  |  |  |  |
| miR-505 | 36.2 | n.s. |  |  |  |  |  |  |
| **miR-93** | **30.1** | **Up** | **0.77** |  | **0.968** | **0.68** | **1.41** | **0.15** |
| (Intercept) |  |  |  |  | -0.998 | 0.23 | -4.17 | 0.000 |

Bold: Eight miRNAs (miR-103, miR-106b, miR-151, miR-17, miR-181a, miR-21, miR-25, miR-93) were significantly upregulated in the refining phase. Abbreviations: Up, Up-regulated; Down, Down-regulated; n.s., Not Significant; AUC, area under curve.

**Supplemental Table 5:** Prediction of serum 8-miR panel and serum SCC-Ag for the differential diagnosis of ESCC from healthy participants in serum training and serum validation cohorts.

|  | **8-miR panel** | | | |  | **SCC-Ag** | |
| --- | --- | --- | --- | --- | --- | --- | --- |
|  | **Training cohort** | **Validation cohort 1** | **Validation cohort 2** | |  | **Validation cohort 2** | |
|  | **(Stage I-IV)** | **(Stage I-III)** | **(Stage I-IV)** | **(Stage I)** |  | **(Stage I-IV)** | **(Stage I)** |
| **AUC** | 0.83 | 0.80 | 0.89 | 0.82 |  | 0.72 | 0.63 |
| **(95% CI)** | (0.79-0.87) | (0.69-0.91) | (0.83-0.94) | (0.69-0.94) |  | (0.59-0.84) | (0.49-0.78) |
| **OR** | 11.2 | 13.0 | 43.2 | 30.08 |  | 22.1 | 6.69 |
| **(95% CI)** | (6.85-18.41) | (4.35-38.56) | (15.59-119.69) | (7.57-119.55) |  | (8.97-54.44) | (2.15-20.76) |
| **Specificity** | 0.76 | 0.60 | 0.86 | 0.90 |  | 0.69 | 0.69 |
| **(95% CI)** | (0.70-0.94) | (0.50-1.00) | (0.78-0.97) | (0.78-0.97) |  | (0.59-0.88) | (0.40-0.83) |
| **Sensitivity** | 0.78 | 0.90 | 0.88 | 0.76 |  | 0.90 | 0.75 |
| **(95% CI)** | (0.57-0.83) | (0.58-0.92) | (0.78-0.92) | (0.60-0.92) |  | (0.80-0.95) | (0.58-0.95) |
| **Accuracy** | 0.78 | 0.85 | 0.87 | 0.85 |  | 0.85 | 0.71 |
| **(95% CI)** | (0.68-0.81) | (0.58-0.92) | (0.80-0.92) | (0.77-0.92) |  | (0.77-0.90) | (0.57-0.81) |
| **NPV** | 0.61 | 0.52 | 0.71 | 0.86 |  | 0.72 | 0.82 |
| **(95% CI)** | (0.49-0.68) | (0.26-0.83) | (0.58-0.81) | (0.79-0.95) |  | (0.55-0.83) | (0.75-0.96) |
| **PPV** | 0.88 | 0.92 | 0.95 | 0.82 |  | 0.89 | 0.58 |
| **(95% CI)** | (0.85-0.96) | (0.90-1.00) | (0.92-0.99) | (0.68-0.95) |  | (0.86-0.95) | (0.45-0.73) |

**Supplemental Table 6.** Comparison of the performance of the circulating miRNA signature against SCC-Ag, CEA, CA72-4, and CYFRA21-1 for non-invasive detection of ESCC across all stages in randomized prospective serum cohorts.

|  | **Training Cohort** | | | | |  | **Validation Cohort** | | | | |
| --- | --- | --- | --- | --- | --- | --- | --- | --- | --- | --- | --- |
|  | **8-miR panel** | **SCC-Ag** | **CEA** | **CA72-4** | **CYFRA21-1** |  | **8-miR panel** | **SCC-Ag** | **CEA** | **CA72-4** | **CYFRA21-1** |
| **AUC** | 0.92 | 0.56 | 0.63 | 0.62 | 0.61 |  | 0.93 | 0.63 | 0.67 | 0.54 | 0.72 |
| **(95% CI)** | (0.87 - 0.96) | (0.47 - 0.65) | (0.55 - 0.71) | (0.44 - 0.80) | (0.45 - 0.78) |  | (0.88 - 0.97) | (0.55 - 0.71) | (0.59 - 0.75) | (0.36 - 0.72) | (0.56 - 0.87) |
| **Odds Ratio** | 42.66 | 4.91 | 3.93 | 0.22 | 3.16 |  | 123.12 | 2.98 | 3.46 | 0.19 | 11.27 |
| **(95% CI)** | (18.09 - 100.62) | (2.07 - 11.65) | (1.88 - 8.21) | (0.06 - 0.87) | (0.87 - 11.51) |  | (42.85 - 353.72) | (1.59 - 5.58) | (1.85 - 6.47) | (0.04 - 1.02) | (2.64 - 48.12) |
| **Specificity** | 0.84 | 0.92 | 0.41 | 0.44 | 0.73 |  | 0.89 | 0.76 | 0.74 | 0.87 | 0.81 |
| **(95% CI)** | (0.78 - 0.97) | (0.58 - 0.99) | (0.29 - 0.68) | (0.25 - 1.00) | (0.33 - 1.00) |  | (0.85 - 0.96) | (0.55 - 0.95) | (0.51 - 0.97) | (0.27 - 1.00) | (0.60 - 1.00) |
| **Sensitivity** | 0.89 | 0.31 | 0.85 | 0.85 | 0.53 |  | 0.93 | 0.49 | 0.54 | 0.44 | 0.72 |
| **(95% CI)** | (0.74 - 0.96) | (0.19 - 0.65) | (0.60 - 0.94) | (0.24 - 1.00) | (0.16 - 0.88) |  | (0.87 - 0.98) | (0.25 - 0.73) | (0.28 - 0.80) | (0.22 - 0.96) | (0.50 - 0.89) |
| **Accuracy** | 0.86 | 0.64 | 0.61 | 0.72 | 0.59 |  | 0.91 | 0.63 | 0.65 | 0.6 | 0.75 |
| **(95% CI)** | (0.82 - 0.91) | (0.59 - 0.69) | (0.56 - 0.69) | (0.46 - 0.84) | (0.38 - 0.79) |  | (0.88 - 0.95) | (0.58 - 0.70) | (0.60 - 0.72) | (0.48 - 0.74) | (0.62 - 0.87) |
| **NPV** | 0.89 | 0.61 | 0.76 | 0.58 | 0.35 |  | 0.94 | 0.64 | 0.66 | 0.46 | 0.57 |
| **(95% CI)** | (0.80 - 0.95) | (0.58 - 0.66) | (0.65 - 0.89) | (0.36 - 1.00) | (0.29 - 0.62) |  | (0.88 - 0.98) | (0.59 - 0.73) | (0.61 - 0.77) | (0.39 - 0.80) | (0.43 - 0.75) |
| **PPV** | 0.84 | 0.76 | 0.55 | 0.76 | 0.85 |  | 0.89 | 0.63 | 0.64 | 0.86 | 0.9 |
| **(95% CI)** | (0.79 - 0.96) | (0.57 - 0.96) | (0.51 - 0.65) | (0.72 - 1.00) | (0.78 - 1.00) |  | (0.84 - 0.95) | (0.55 - 0.82) | (0.56 - 0.90) | (0.69 - 1.00) | (0.81 - 1.00) |

**Supplemental Table 7.** Benchmark the performance of the circulating miRNA signature against SCC-Ag and CEA for non-invasive detection of stage I ESCC in randomized prospective serum cohorts.

|  | **Training Cohort** | | |  | **Validation Cohort** | | |
| --- | --- | --- | --- | --- | --- | --- | --- |
|  | **8-miR panel** | **SCC-Ag** | **CEA** |  | **8-miR panel** | **SCC-Ag** | **CEA** |
| AUC | 0.97 | 0.65 | 0.61 |  | 0.89 | 0.62 | 0.66 |
| (95% CI) | (0.93 - 1.00) | (0.49 - 0.82) | (0.45 - 0.76) |  | (0.77 - 1.00) | (0.43 - 0.81) | (0.46 - 0.85) |
| Odds Ratio | 121 | 0.25 | 3.86 |  | 106.8 | 0.26 | 20.14 |
| (95% CI) | (13.80 - 1061.12) | (0.07 - 0.85) | (0.98 - 15.14) |  | (12.54 - 909.71) | (0.08 - 0.85) | (4.59 - 88.48) |
| Specificity | 0.92 | 0.8 | 0.56 |  | 0.90 | 0.77 | 0.96 |
| (95% CI) | (0.77 - 0.98) | (0.19 - 0.93) | (0.24 - 0.79) |  | (0.85 - 0.97) | (0.20 - 0.95) | (0.24 - 1.00) |
| Sensitivity | 0.92 | 0.5 | 0.75 |  | 0.92 | 0.54 | 0.46 |
| (95% CI) | (0.92 - 1.00) | (0.33 - 1.00) | (0.50 - 1.00) |  | (0.77 - 1.00) | (0.23 - 1.00) | (0.23 - 1.00) |
| Accuracy | 0.92 | 0.77 | 0.58 |  | 0.9 | 0.74 | 0.9 |
| (95% CI) | (0.80 - 0.98) | (0.28 - 0.88) | (0.32 - 0.77) |  | (0.86 - 0.96) | (0.29 - 0.89) | (0.33 - 0.95) |
| NPV | 0.99 | 0.93 | 0.95 |  | 0.99 | 0.93 | 0.93 |
| (95% CI) | (0.99 - 1.00) | (0.92 - 1.00) | (0.92 - 1.00) |  | (0.97 - 1.00) | (0.90 - 1.00) | (0.90 - 1.00) |
| PPV | 0.58 | 0.24 | 0.18 |  | 0.55 | 0.23 | 0.6 |
| (95% CI) | (0.35 - 0.88) | (0.13 - 0.47) | (0.13 - 0.27) |  | (0.43 - 0.80) | (0.14 - 0.54) | (0.15 - 1.00) |

**Supplemental Table 8.** Univariate and multivariate analyses of the circulating miRNA signature with SCC-Ag, CEA, CA72-4, and CYFRA21-1 for non-invasive detection of ESCC across all stages in randomized prospective serum cohorts.

|  | **Training Cohort** | | | |  | **Validation Cohort** | | | |
| --- | --- | --- | --- | --- | --- | --- | --- | --- | --- |
|  | **Univariable** | | **Multivariable** | |  | **Univariable** | | **Multivariable** | |
|  | **OR (95% CI)** | ***P*** | **OR (95% CI)** | ***P*** |  | **OR (95% CI)** | ***P*** | **OR (95% CI)** | ***P*** |
| **8-miR** | 134.33 (45.81 - 393.96) | 1.50E-13 | 4.45 (2.09 - 18.42) | 0.0037 |  | 50.93 (21.26 - 122.00) | 5.70E-12 | 13.60 (2.78 - 912.46) | 0.048 |
| **Sex** | 4.28 (2.03 - 9.05) | 0.00014 | 6.76 (0.44 - 298.98) | 0.21 |  | 3.44 (1.55 - 7.60) | 0.0023 | 85.05 (0.08 - 162.15) | 0.29 |
| **Age** | 2.40 (1.32 - 4.36) | 0.00028 | 1.07 (0.95 - 1.25) | 0.32 |  | 7.67 (2.81 - 20.92) | 0.0011 | 0.98 (0.47 - 1.02) | 0.94 |
| **SCC** | 5.88 (2.48 - 13.91) | 0.0044 | 1.13 (0.25 - 5.57) | 0.87 |  | 2.58 (1.41 - 4.71) | 0.041 | 1.69 (0.91 - 136507.34) | 0.89 |
| **CEA** | 2.53 (1.37 - 4.67) | 0.0081 | 0.86 (0.37 - 1.92) | 0.71 |  | 4.69 (2.45 - 8.98) | 0.00077 | 0.39 (0.04 - 1.63) | 0.28 |
| **CA72-4** | 0.46 (0.13 - 1.59) | 0.86 | 1.11 (0.70 - 1.96) | 0.7 |  | 0.15 (0.03 - 0.68) | 0.1 | 0.81 (0.32 - 1.17) | 0.4 |
| **CYFRA21-1** | 7.56 (1.87 - 30.58) | 0.069 | 1.01 (0.45 - 2.83) | 0.99 |  | 4.17 (1.04 - 16.73) | 0.3 | 0.82 (0.04 - 9.15) | 0.86 |

**Supplemental Table 9.** Univariate and multivariate analyses of the circulating miRNA signature with SCC-Ag and CEA for non-invasive detection of stage I ESCC in randomized prospective serum cohorts.

|  | **Training Cohort** | | | |  | **Validation Cohort** | | | |
| --- | --- | --- | --- | --- | --- | --- | --- | --- | --- |
|  | **Univariable** | | **Multivariable** | |  | **Univariable** | | **Multivariable** | |
|  | **OR (95% CI)** | ***P*** | **OR (95% CI)** | ***P*** |  | **OR (95% CI)** | ***P*** | **OR (95% CI)** | ***P*** |
| **8-miR** | 190.00 (21.71 - 1662.60) | 1.00E-05 | 3.56 (2.15 - 7.19) | 2.40E-05 |  | 56.41 (3.19 - 998.16) | 0.0034 | 1.64 (1.21 - 2.41) | 0.0043 |
| **Sex** | 1.77 (0.53 - 5.96) | 0.35 | 0.36 (0.05 - 2.58) | 0.3 |  | 1.64 (0.33 - 8.21) | 0.55 | 1.29 (0.21 - 11.41) | 0.79 |
| **Age** | 0.20 (0.06 - 0.63) | 0.74 | 1.00 (0.92 - 1.09) | 0.98 |  | 3.38 (0.82 - 13.89) | 0.3 | 1.06 (0.98 - 1.16) | 0.2 |
| **SCC** | 0.11 (0.04 - 0.37) | 0.3 | 0.98 (0.17 - 6.01) | 0.98 |  | 1.54 (0.37 - 6.35) | 0.41 | 0.41 (0.04 - 2.30) | 0.42 |
| **CEA** | 3.22 (0.96 - 10.78) | 0.068 | 1.69 (0.96 - 3.21) | 0.084 |  | 9.76 (0.55 - 172.14) | 0.11 | 1.02 (0.58 - 1.71) | 0.94 |

**Supplemental Table 10.** Results of cost-effectiveness analysis for non-invasive screening for Chinese men in China (> 40 years old)

|  | **Non-invasive screening program** | **No-screening** |
| --- | --- | --- |
| Cohort size | 100000 | 100000 |
| Total number of cancer patients in the cohort | 5920 | 5920 |
| Compliance | 45% | 10% |
| Stage of diagnosis (Stage 1; Stage 2; Stage 3; Stage4) | 15.50%; 30.44%; 28.61%; 21.50% | 7.56%; 34.65%; 31.37% 19.24% |
| Average cost of diagnosis | 553.6 CNY | 303.0 CNY |
| **Results of cost-effectiveness analysis** | | |
| Total cost | 921.8M | 893.2M |
| Total QALY | 294.5K | 294.3K |
| Cost difference | 269.9 |  |
| Effect difference | 0.017 |  |
| Cost of saving 1 QALY | 15800.4 CNY/QALY |  |

CNY: Chinese Yuan; QALY: quality adjusted life year.

**Supplemental Table 11.** Base-case values in cost-effectiveness modeling

|  | **Value** | **Ref.** |
| --- | --- | --- |
| **Cost** |  |  |
| miRNA assay | 300 | Estimated |
| Endoscopy | 1620 | Yang J et al. Asian Pac J Cancer Prev. 2011,12(5):1245-1250. |
| Biopsy | 1292 |  |
| Stage 1 treatment | 34460 | Guo LW et al. Chinese Journal of Cancer.2017,36:73. |
| Stage 2 treatment | 39302 |  |
| Stage 3 treatment | 40353 |  |
| Stage 4 treatment | 37432 |  |
| Follow-up examinations | 695 | Yang J et al. World J Gastroenterol 2012,18(20):2493-2501. |
| Staging Investigation | 2000 | Estimated using in-house records |
|  |  |  |
| **Cancer cases in high risk population** | 5.92% | Wei WQ et al. J Clin Oncol.2015,33(17):1951-1957 |
|  |  |  |
| **Incidence of cancer** | (1/100000) |  |
| 40-44 | 1.31 | 2017 China Cancer Registry Annal Report |
| 45-49 | 4.26 |  |
| 50-54 | 9.31 |  |
| 55-59 | 20.51 |  |
| 60-64 | 36.44 |  |
| 65-69 | 58.45 |  |
| 70-74 | 80.97 |  |
| 75-79 | 104.30 |  |
| 80-84 | 126.20 |  |
|  |  |  |
| **5-yr recurrence rate of cancer by stage** | |  |
| Stage 1 | 22.20% | Zhou Sha et al. Ann surg oncol.2018,25:3639-3646. |
| Stage 2 | 38.70% |  |
| Stage 3 | 68.20% |  |
|  |  |  |
| **Utility Values** |  |  |
| Cancer | 0.75 | Shi JF et al. Poster Abstracts |
| Cured | 0.85 | Estimated |
